# Photocatalytic and Gas Sensitive Multiwalled Carbon Nanotube/TiO_2_-ZnO and ZnO-TiO_2_ Composites Prepared by Atomic Layer Deposition

**DOI:** 10.3390/nano10020252

**Published:** 2020-01-31

**Authors:** László Péter Bakos, Nóra Justh, Ulisses Carlo Moura da Silva Bezerra da Costa, Krisztina László, János László Lábár, Tamás Igricz, Katalin Varga-Josepovits, Pawel Pasierb, Elina Färm, Mikko Ritala, Markku Leskelä, Imre Miklós Szilágyi

**Affiliations:** 1 Department of Inorganic and Analytical Chemistry, Budapest University of Technology and Economics, Szent Gellért tér 4., H-1111 Budapest, Hungary; justh.nora@gmail.com (N.J.); ulissesccosta@gmail.com (U.C.M.d.S.B.d.C.); imre.szilagyi@mail.bme.hu (I.M.S.); 2 Department of Physical Chemistry and Materials Science, Budapest University of Technology and Economics, P.O. Box 92, H-1521 Budapest, Hungary; klaszlo@mail.bme.hu; 3 Institute for Technical Physics and Materials Science, Research Centre of Energy, Hungarian Academy of Sciences, Konkoly–Thege út 29–33., H-1121 Budapest, Hungary; labar.janos@energia.mta.hu; 4 Department of Organic Chemistry and Technology, Budapest University of Technology and Economics, Budafoki út 8., H-1111 Budapest, Hungary; igricz.tamas@gmail.com; 5 Department of Atomic Physics, Budapest University of Technology and Economics, Budafoki út 8., H-1111 Budapest, Hungary; flip@eik.bme.hu; 6 Department of Inorganic Chemistry, AGH University of Science and Technology, Mickiewicza 30., 30-059 Kraków, Poland; ppasierb@agh.edu.pl; 7 ASM Microchemistry Oy, Pietari Kalmin katu 1F2, FI-00560 Helsinki, Finland; elina.farm@helsinki.fi; 8 Department of Chemistry, University of Helsinki, P.O. Box 55, FI-00014 Helsinki, Finland; mikko.ritala@helsinki.fi (M.R.); markku.leskela@helsinki.fi (M.L.)

**Keywords:** carbon nanotubes, titanium dioxide, zinc oxide, atomic layer deposition, photocatalysis, gas sensing

## Abstract

TiO_2_ and ZnO single and multilayers were deposited on hydroxyl functionalized multi-walled carbon nanotubes using atomic layer deposition. The bare carbon nanotubes and the resulting heterostructures were characterized by TG/DTA, Raman, XRD, SEM-EDX, XPS, TEM-EELS-SAED and low temperature nitrogen adsorption techniques, and their photocatalytic and gas sensing activities were also studied. The carbon nanotubes (CNTs) were uniformly covered with anatase TiO_2_ and wurtzite ZnO layers and with their combinations. In the photocatalytic degradation of methyl orange, the most beneficial structures are those where ZnO is the external layer, both in the case of single and double oxide layer covered CNTs (CNT-ZnO and CNT-TiO_2_-ZnO). The samples with multilayer oxides (CNT-ZnO-TiO_2_ and CNT-TiO_2_-ZnO) have lower catalytic activity due to their larger average densities, and consequently lower surface areas, compared to single oxide layer coated CNTs (CNT-ZnO and CNT-TiO_2_). In contrast, in gas sensing it is advantageous to have TiO_2_ as the outer layer. Since ZnO has higher conductivity, its gas sensing signals are lower when reacting with NH_3_ gas. The double oxide layer samples have higher resistivity, and hence a larger gas sensing response than their single oxide layer counterparts.

## 1. Introduction

Carbon nanotubes (CNTs) have attracted great interest for application in diverse fields owing to their unique chemical and physical properties. These fields include conductive and high-strength composites, energy storage and energy conversion devices, sensors, field emission displays and radiation sources, hydrogen storage media and heterogeneous catalysis [1,2,3,4,5,6,7,8,9,10,11]. TiO_2_ and ZnO are widely researched materials in various areas, including photocatalytic wastewater treatment and hydrogen production, solar cells and gas sensing [12,13,14,15,16,17,18,19,20,21]. CNTs have been successfully used as catalyst support materials. As photocatalyst carriers they can increase the separation and migration of photogenerated electrons and holes [22,23,24]. Carbon nanotubes and semiconducting metal oxides have been studied in gas sensing applications, because adsorption and desorption of gas molecules on the surface results in charge transfer, which leads to change in electric resistance. The detection sensitivity to gases depends on many factors; e.g., grain size, surface composition and morphology and the target gas [25,26,27,28,29,30,31]. 

CNT-TiO_2_ and CNT-ZnO composites can be prepared by various methods; e.g., hydrothermal synthesis, sputtering, chemical vapor deposition (CVD) and atomic layer deposition (ALD) [32,33,34]. Among them, ALD has the advantage that it allows the coating of nanostructures in a conformal and homogenous way, with a precise control of the thickness of the deposited film at the nanoscale by successive, self-terminating, gas–surface half-reactions [35,36,37,38,39,40,41]. 

Different metal oxides were already deposited on CNTs by ALD, e.g., Al_2_O_3_, SnO_2_, V_2_O_4_, HfO_2_, Fe_2_O_3_, TiO_2_ and ZnO, and in some cases they were studied in gas sensing or photocatalysis applications [42,43,44,45,46,47]. Previously, it was also studied how changing the core and shell materials influence the photocatalytic activity and gas sensing properties of TiO_2_-ZnO and ZnO-TiO_2_ core-shell nanofibers prepared by electrospinning and ALD [48,49]. However, to the best of our knowledge, multilayer oxide coatings have not been grown on CNTs to obtain photocatalytic and gas sensitive core-shell nanocomposites. 

In our research, ALD TiO_2_ and ZnO metal oxide layers were grown on multi-walled CNTs, which were functionalized with hydroxyl groups (CNT-OH) serving as ALD nucleation sites. Both single and double oxide layer coated CNT composites, i.e., CNT-TiO_2_ and CNT-ZnO, and CNT-TiO_2_-ZnO and CNT-ZnO-TiO_2_ samples, were obtained. The name indicates the sequence of the oxide ALD processes. The carbon nanotubes and the resulting CNT-metal oxide composite materials were characterized using TG/DTA, Raman spectroscopy, XRD, XPS, SEM-EDX, TEM-EELS-SAED and low temperature N_2_ adsorption. Finally, the activities of the as-prepared samples as photocatalysts and NH_3_ gas sensors were studied.

## 2. Materials and Methods 

### 2.1. Sample Preparation

Hydroxyl functionalized multi-walled carbon nanotubes with external diameters of 30–50 nm were used as obtained (Cheap Tubes, Cambridgeport, VT, USA). 

Atomic layer deposition of TiO_2_ and ZnO was performed in an ASM F-120 ALD reactor by the reaction of TiCl_4_ and (C_2_H_5_)_2_Zn with H_2_O, respectively. The reactor was operated at a pressure of about 5 mbar using nitrogen as the carrier and purge gas. For one batch, 50 mg substrate powder was placed in a folded steel mesh into the reactor. The parameters of the ALD processes are summarized in Table 1. Each time, 12 nm thick films were grown. The thicknesses were approximated by X-ray reflectometry (Bruker D8 Advance) on Si reference wafers that were coated together with CNTs.

### 2.2. Characterization

The details of the instruments and characterization methods used can be found in the Supplementary Materials. TG/DTA curves were measured in inert (N_2_) and oxidizing (air) conditions with a linear temperature program (10 °C min^−1^). To get structure data, Raman, XRD and SAED studies were done. To see the morphologies of the samples, SEM and TEM images were taken. The elemental compositions and bonding states and were investigated with XPS, EDX and EELS. Nitrogen adsorption was utilized to measure the specific surface area. The photocatalytic activity was investigated by decomposing an aqueous solution of methyl orange under UV light irradiation (spectrum: Figure S1), and P25 Degussa TiO_2_ was used for reference material. NH_3_ gas sensing properties were tested at different concentrations and temperatures (setup: Figures S27–S28). 

## 3. Results and Discussion

### 3.1. TG/DTA

**Figure 1:**
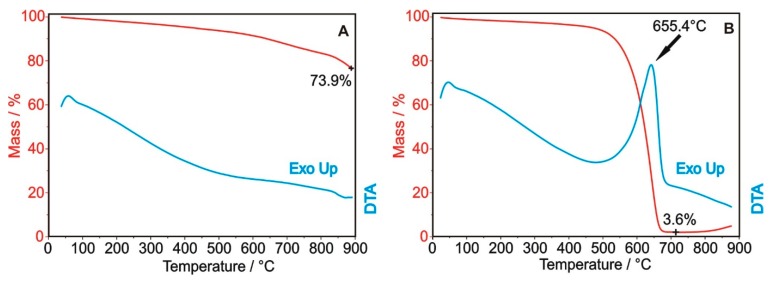
TG/DTA measurements of the OH-functionalized carbon nanotubes in nitrogen (**A**) and air atmospheres (**B**).

From the thermal analysis in nitrogen (Figure 1A), it can be seen that the bare OH-functionalized carbon nanotubes lose 26.1% of the initial mass, accompanied by an endothermic process. The decomposition is not significant at the early stages; thus, the ALD process can be safely performed at 200 °C for ZnO and at 250 °C for TiO_2_ without damaging the carbon nanotube substrates. In Figure 1B, the thermal analysis in air shows that the exothermic combustion of the carbon nanotubes starts around 500 °C, leaving only 3.6% residual mass at 700 °C. The residue is the catalyst used for CNT preparation, which upon further annealing in air at 900 °C gets oxidized. 

### 3.2. Raman Spectroscopy

**Figure 2:**
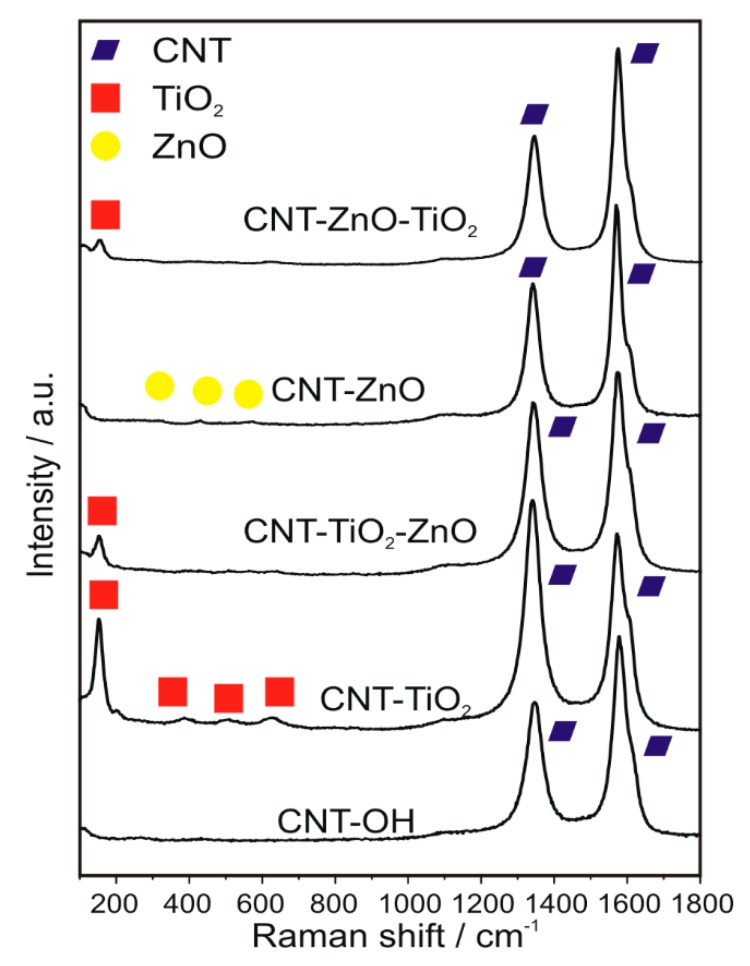
Raman spectra of the samples.

The D and G peaks of the carbon are present in all samples (Figure 2). In the TiO_2_ containing samples, the most intensive additional band is the TiO_2_ (anatase) at 141 cm^−1^. The further characteristic peaks of TiO_2_ at 400, 516 and 637 cm^−1^ are the most obvious in the CNT-TiO_2_ sample [50]. The bands of the ZnO are also detectable at 320, 428 and 569 cm^−1^, but they are much weaker compared to the TiO_2_ signals. The presence of both TiO_2_ and ZnO was also verified later by XRD. In the CNT-double oxide samples both oxide signals show a reduced intensity compared with the CNTs coated by any of the single oxide layers.

### 3.3. Powder XRD

**Figure 3:**
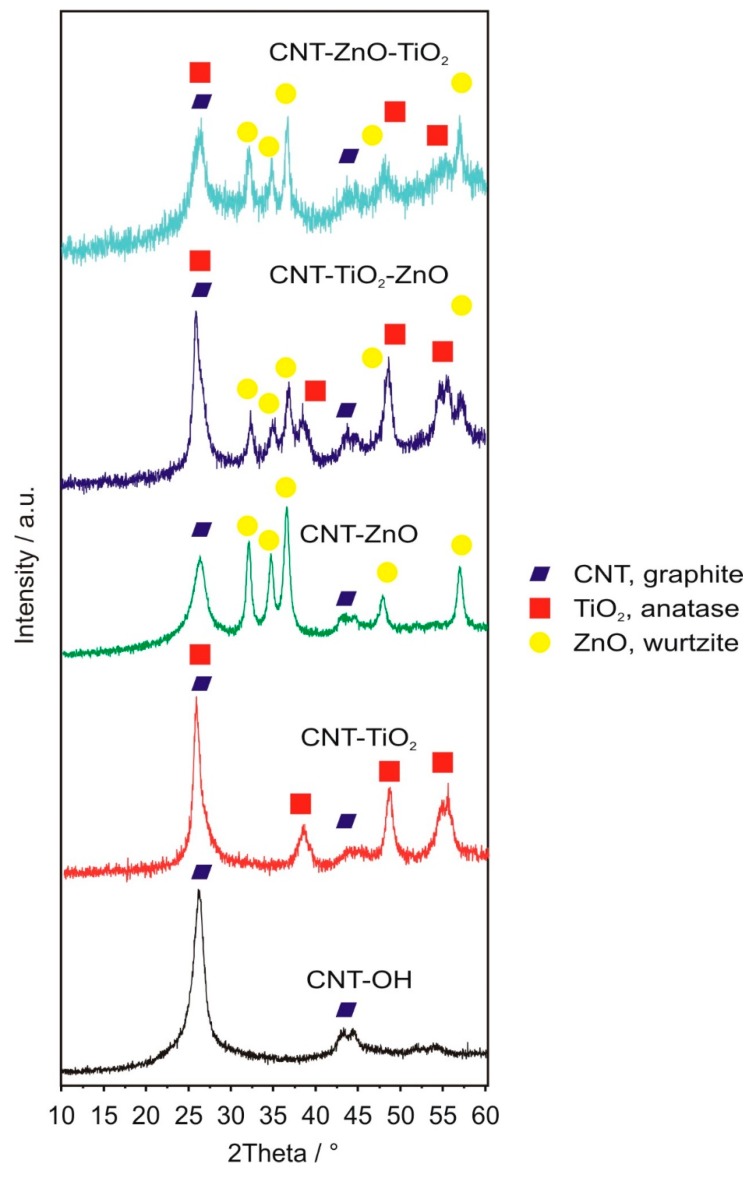
XRD diffractograms of the samples and the assignation of the peaks.

XRD diffractograms (Figure 3) confirm the presence of the semiconductor oxides deposited on the carbon nanotubes. In the case of the bare carbon nanotubes, the peaks are characteristic of the rolled graphite layers in the multiwalled CNTs (ICDD 01-71-4630). The as-grown oxide materials are crystalline; i.e., TiO_2_ is present as the anatase (ICDD 01-075-2546), and ZnO as the wurtzite (ICDD 01-080-4199), corroborating Raman spectra. Similarly to the Raman results, the oxide signals are weaker in the case of the double oxide layer coatings, than in the single oxide layer coated samples. This might imply an interaction, e.g., interdiffusion between the oxide layers, which may occur during the ALD growth of the second oxide layer. This could reduce the crystalline order of the layers, and thus, the XRD and Raman peak intensities.

### 3.4. SEM and TEM

**Figure 4:**
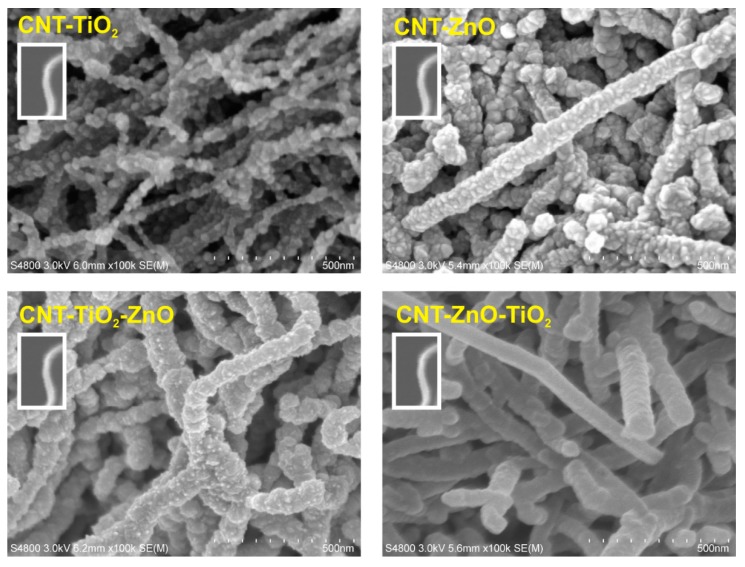
SEM images of the samples. In the white frame, the SEM picture of an uncoated CNT-OH is shown at the same magnification for reference.

Figure 4 portraits SEM images of the samples after the ALD processes. The as-grown oxide layers are clearly visible on the surface of the CNTs. In the ALD reactions, both ZnO and TiO_2_ nucleate as grains, and then coalesce to form a continuous layer. Since ZnO crystallizes easier than TiO_2_ during the ALD depositions, it seems to form larger grains than TiO_2_. In the case of the double oxide layers, the oxide coating on the CNTs becomes thicker and smoother. Based on the resolution of the SEM images, the smoothest surface was obtained in the CNT-ZnO-TiO_2_ sample. The approximate diameters for the samples were around 35 nm for the CNT-OH, 50 nm for the CNT-TiO_2_ and 70 nm for all the other composites.

**Figure 5:**
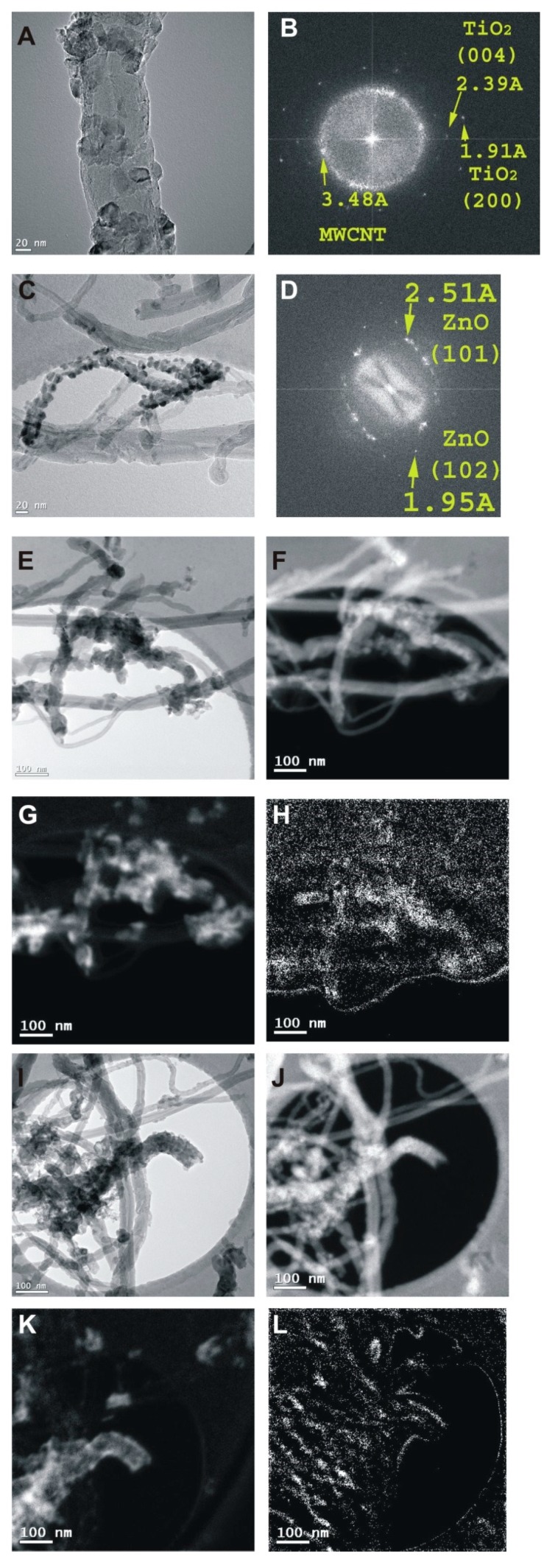
TEM (**A**) and FFT (**B**) of images of the CNT-TiO_2_ sample; TEM (**C**) and FFT (**D**) of images of the CNT-ZnO sample; TEM (**E**) image and C (**F**), Ti (**G**) and Zn (**H**) EELS maps of the CNT-TiO_2_-ZnO sample; TEM (**I**) image and C (**J**), Ti (**K**) and Zn (**L**) EELS maps of the CNT-ZnO-TiO_2_ sample_._ The FFT of a TEM image has the same informational content as a SAED pattern (and is similar in appearance) from the same area.

The TEM images of the CNT-TiO_2_ and the CNT-ZnO samples (Figure 5A and Figure 5C, respectively) reveal the metal oxide layers composed of oxide nanoparticles on the carbon nanotubes. The electron diffraction patterns of the CNT-TiO_2_ (Figure 5B) and CNT-ZnO (Figure 5D) samples corroborate the XRD and Raman measurements, revealing that the oxides are present as anatase and wurtzite, respectively. The TEM images of the double oxide layer coated samples (Figure 5E–I) show again that the CNTs have thicker oxide shells. The EELS maps of the C, Ti and Zn of the CNT-TiO_2_-ZnO and CNT-ZnO-TiO_2_ samples present the carbon core morphology surrounded by the Ti and Zn oxide layers. It is shown that the oxide layers cover the CNTs uniformly.

### 3.5. EDX and XPS

EDX and XPS measurements also confirm that the deposition of semiconductor oxide layers was successful in the case of all samples (Table 2 and Figures S2–S27). The XPS energies of 2p_3/2_ peaks in all samples at 459.4 eV and at 1024.0 eV for the Ti and Zn, respectively, indicate that they are present as TiO_2_ and ZnO [51,52].

These two methods complement each other, as XPS provides information mainly about the top 5–10 nm layer, while the information depth of EDX is ca. 0.5–1 μm. That is, XPS provides data about the very surface of the composites, and EDX, rather, about the bulk average. Accordingly, XPS measures 5−10 times higher concentration for Ti and Zn present on the surface of the CNT-oxide composite samples than EDX. 

Based on the peak deconvolution (Table 3), the O1s peak of the bare CNT-OH sample contains the oxygen in two different states; i.e., the peak at 530.3 eV corresponds to the oxygen in physically adsorbed water and possible surface carbonate/carboxyl groups, while the peak at 533.0 eV is the oxygen in the OH group and chemisorbed water. The O1s peak of the coated samples at 530.8−531.2 eV comes mostly from the oxygen in the metal oxides. Compared to the bare CNT-OH, the intensity of this peak increases gradually when single and double oxide layers are grown, and simultaneously, the intensity of the OH peak between 532.2 and 532.7 decreases because the ALD reactions use up a significant amount of the OH groups [48]. 

Carbon is present in three different forms. The peak at 284.0 eV corresponds to the C–C bond, while the peak between 285.1 and 285.3 eV is related to structural defects, attributed to C atoms no longer in the regular tubular structure. The ratio of the defect related carbon increases after the ALD reactions, while the intensity of the C–C peak decreases. The signal in the 289.4−289.8 eV range is related to the bond between C and O. Its intensity does not change significantly after the ALD processes, since most of the C–OH moieties are replaced by C–O–metal bonds [53].

### 3.6. Nitrogen Adsorption

The surface areas from the nitrogen adsorption measurements are shown in Table 4. The bare CNT-OH has the largest apparent surface area. After coating with a single metal oxide layer, its value decreases, and by applying a second layer, it decreases further. As the densities of the deposited oxides are significantly greater than that of the carbon nanotubes, after the ALD film growth, the average density of each sample increases. This is partly responsible for the decreasing specific surface area.

### 3.7. Photocatalysis

**Figure 6:**
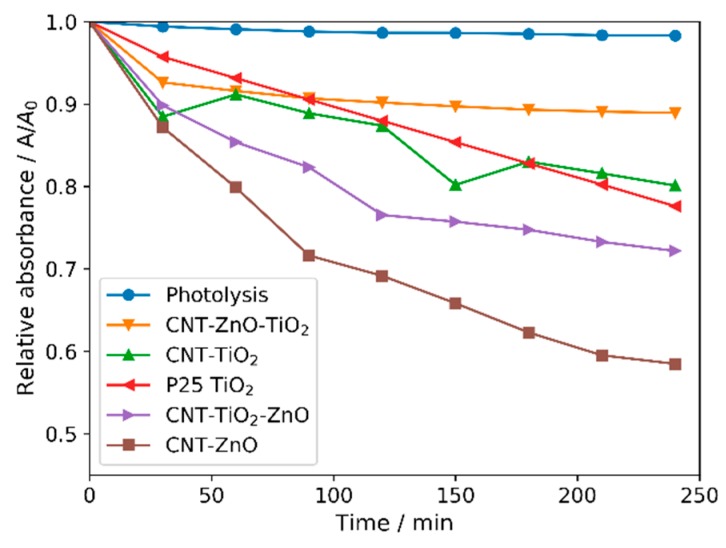
Photocatalytic activities of the samples.

**Figure 7:**
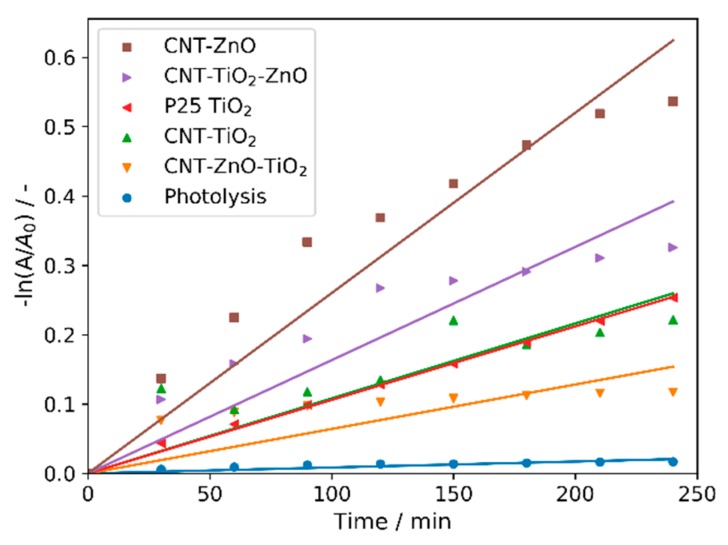
Pseudo first order linear fitting of the photocatalysis.

All CNT-metal oxide composites possess photocatalytic activity—decomposing methyl orange (Figure 6 and Figure 7). The CNT-ZnO sample has the best photocatalytic performance, also confirmed by the apparent reaction rate constants of the photocatalysis (Table 5) [46]. Compared to this, a significantly lower activity was observed when CNTs were coated with a TiO_2_ layer. When the second oxide layers are grown onto the CNT-single oxide layer composites, the photocatalytic activity decreases in the case of both CNT-ZnO and CNT-TiO_2_ substrates. The reason for this is that when the amount of heavier oxides increases compared to the lighter carbon core, the average density increases, and the surface area introduced by the 1 mg catalyst sample into the photocatalytic tests is decreased. In the case of the double layered samples, the photocatalytic results are better when ZnO is the outer layer. This is in line with the observation that the CNT-ZnO is a better photocatalyst than CNT-TiO_2_. In fact, even the CNT-TiO_2_-ZnO sample has higher activity than the CNT-TiO_2_ sample. 

In case of the monolayer coated samples, the CNT core as an electron acceptor serves as a sink for the photogenerated electrons, while the holes stay in the metal oxides. This process slows the electron-hole recombination rate, thereby increasing the photocatalytic activity, as the holes can initialize the radical formation more efficiently compared to the bare metal oxides. Moreover, the metal–oxygen–carbon bonds act as sensitizers by narrowing the band gap [54,55]. Despite these effects, CNT-TiO_2_ still performed worse than the reference P25 TiO_2_, while CNT-ZnO was by far the most effective photocatalyst. For the double-layer coated composites, the obtained results correspond to former studies on ZnO-TiO_2_ and TiO_2_-ZnO core/shell nanofibers prepared by electrospinning and ALD [48,56]. The electron-hole pairs are generated mainly in the shell layers under illumination; the holes prefer the ZnO while the electrons migrate to the TiO_2_. For photocatalysis the outer layer is more important, since it is in physical contact with the material to be decomposed. Therefore, photogenerated electrons are mostly responsible for the photocatalytic activity in the case of the CNT-ZnO-TiO_2_ composite, while the photocatalysis in the presence of the CNT-TiO_2_-ZnO composite is controlled by the holes. To generate reactive species, the electrons have to react with oxygen, which is not abundant in the solution, while the holes react with water to produce OH radicals; thus, the ZnO outer layer is preferred. Based on previous results and our present results, higher photocatalytic activity is observed when ZnO is the outer layer than with TiO_2_ being the outer one, and it is even higher than with the conventional P25 reference TiO_2_. This can be explained by the surface chemistry and the intrinsic characteristics of the photogenerated charge carriers. The amount of the chemisorbed oxygen at the lattice defect sites (532.3 eV peak on XPS, Table 3) for CNT-TiO_2_-ZnO is 1.3 times higher than for the CNT-ZnO-TiO_2_. ZnO may enhance the hole capture process through O defects, resulting in a lower binding energy for adsorbates, and a comparatively lower mobility of the holes in its valence band. 

### 3.8. Gas Sensing

**Figure 8:**
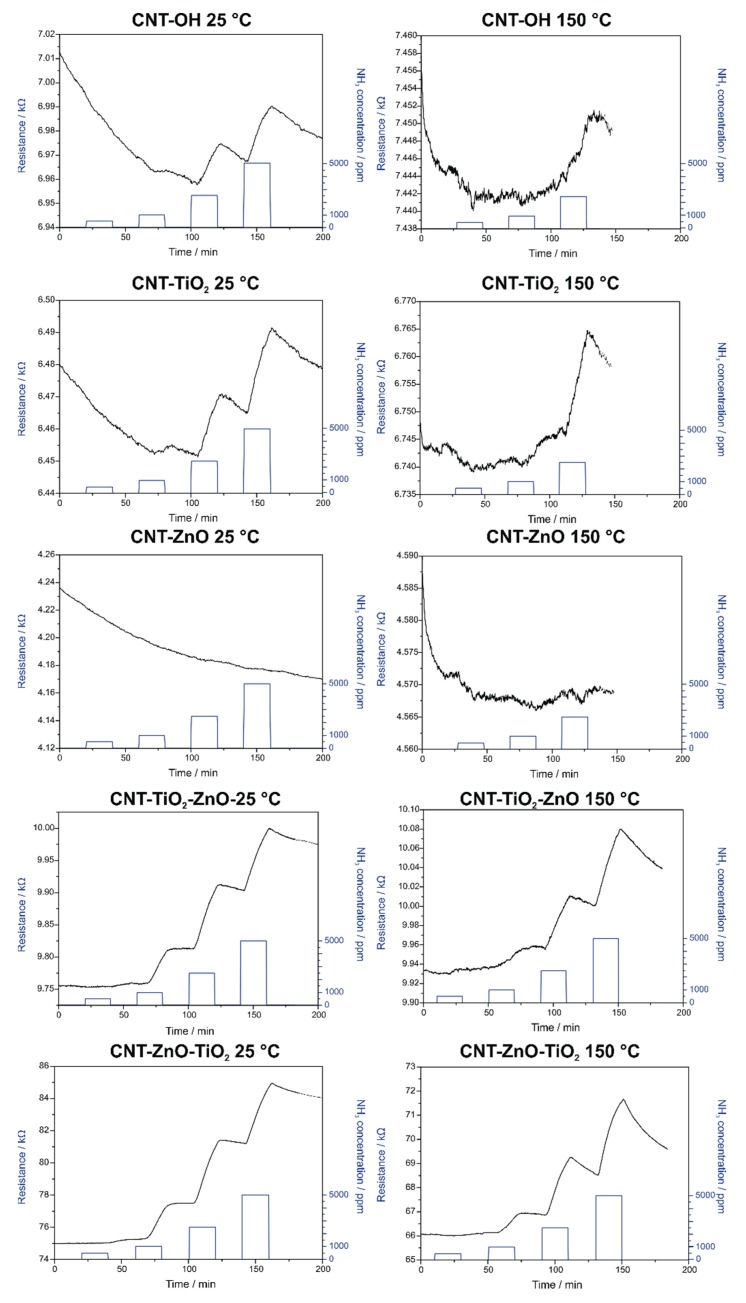
NH_3_ gas sensing measurements of the various samples.

The gas sensing results are shown in Figure 8. An n-type NH_3_ gas response is visible in all cases. The gas sensing activity is better with all samples at 25 °C than at 150 °C, because the conductivity of the semiconductors layers increases with temperature, and hence the gas responses are lower. There are small delays in the responses and recoveries for all samples at all detected gas concentrations due to the gas sensing setup volume. 

The CNT-OH sample shows a small sensitivity at room temperature, which becomes even lower at 150 °C. When ZnO is deposited on the CNTs, the surface conductivity seems to increase, which is reflected by the decreased base resistivity, and also by the fact that that hardly any gas sensing signal is observed for this sample, even at 25 °C. When CNTs are covered with a single layer of TiO_2_ by ALD, the room temperature response increases compared to that with CNT-OH, and the gas sensing property is also more pronounced at 150 °C. The resistivity of the ALD TiO_2_ is often low [57,58]. Unlike the single layer oxide coatings, the CNT-multilayer oxide composites have a considerably better gas sensing performance, due to the increased resistivity of the thicker oxide shells. Because of the two layers, there is contact resistance between them, the coverage is smoother and there are less holes in the coating. In the case of these samples, it is also obvious that the order of the layers has a significant impact on the gas sensing properties. When TiO_2_ is the outer layer, the response is around forty times greater at both temperatures, compared to when ZnO is the outer layer. This is explained by the lower conductivity of TiO_2_ compared to ZnO. The interaction with the NH_3_ gas results in a larger change in its surface resistance [59]. Therefore, in contrast to photocatalysis, for which a ZnO outer layer is more beneficial, in the case of gas sensing, it is preferable to have TiO_2_ as the outer layer.

## 4. Conclusions

TiO_2_ and ZnO single and multilayers have been successfully deposited on hydroxyl functionalized multi-walled carbon nanotubes. CNT-TiO_2_, CNT-ZnO, CNT-TiO_2_-ZnO and CNT-ZnO-TiO_2_ core/shell nanocomposites are obtained. TG/DTA curves reveal that CNT-OH is thermally stable at the ALD temperatures; i.e., at 250 °C and 200 for TiO_2_ and ZnO, respectively. EDX, XPS and Raman spectroscopy confirm the presence of the deposited oxides on the CNTs. The as-grown metal oxide layers are crystalline; i.e., TiO_2_ is present as anatase and ZnO as wurtzite. SEM and TEM images show that the TiO_2_ and ZnO nucleate as particles on CNTs, which merge to form uniform and continuous layers. Due to the presence of heavier oxide coating on the lighter carbon cores, the specific surface area of the nanocomposites decreases compared to the bare CNT-OH sample. The composites possess significant photocatalytic activity in decomposing methyl orange dye under UV light irradiation. The most beneficial structure is when ZnO is the outer layer, both in the case of single and double oxide layer covered CNTs. The samples with multilayer oxides have lower activity due to their larger average density and lower specific surface area. In contrast to photocatalysis, in gas sensing the advantageous structure is the one where TiO_2_ is the outer layer, since ZnO has higher conductivity, and thus, its gas-sensing signals are lower than those of TiO_2_ when interacting with NH_3_ gas. Both the resistivity and the gas sensing signals are higher in multilayer oxide coated CNTs than either in CNT-TiO_2_ or CNT-ZnO.

## Figures and Tables

**Table 1:** ALD process parameters.

Deposited Oxide	Temperature/°C	Number of Cycles	Pulse Times/s			
			Metallic Precursor	N_2_ Purge	Water	N_2_ Purge
TiO_2_	250	250	1.5	30	3	30
ZnO	200	120	3	30	3	30

**Table 2:** Elemental composition from EDX and XPS measurements.

Sample	EDX				XPS			
	Atomic %							
	C	O	Ti	Zn	C	O	Ti	Zn
CNT-OH	96.0	4.0			96.3	3.7		
CNT-TiO_2_	82.0	16.2	1.8		74.7	18.8	6.5	
CNT-ZnO	92.2	7.0		0.7	82.4	8.6		9.0
CNT-TiO_2_-ZnO	73.5	21.1	4.8	0.5	71.1	16.7	5.0	7.2
CNT-ZnO-TiO_2_	58.2	34.3	6.6	0.9	60.0	26.2	8.4	5.4

**Table 3:** Deconvolutions of the O1s and the C1s peaks from XPS measurements.

**Deconvolution of the O1s Peak**									
**CNT-OH**		**CNT-TiO_2_**		**CNT-TiO_2_-ZnO**		**CNT-ZnO**		**CNT-ZnO-TiO_2_**	
**Position/eV**	**At. %**	**Position/eV**	**At. %**	**Position/eV**	**At. %**	**Position/eV**	**At. %**	**Position/eV**	**At. %**
530.3	18.5	530.8	72.5	530.9	65.2	531.2	43.7	530.9	73.5
533.0	81.5	532.3	27.5	532.3	34.8	532.7	56.3	532.2	26.5
**Deconvolution of the C1s Peak**									
**CNT-OH**		**CNT-TiO_2_**		**CNT-TiO_2_-ZnO**		**CNT-ZnO**		**CNT-ZnO-TiO_2_**	
**Position/eV**	**At. %**	**Position/eV**	**At. %**	**Position/eV**	**At. %**	**Position/eV**	**At. %**	**Position/eV**	**At. %**
284.0	72.6	284.0	61.8	284.0	66.8	284.0	63.5	284	61.1
285.3	19.7	285.1	30.8	285.3	25.1	285.2	27.6	285.2	29.5
289.4	7.7	289.5	7.4	289.6	8.1	289.8	8.9	289.5	9.4

**Table 4:** The measurements of the specific surface areas of the samples.

Sample	CNT-OH	CNT-TiO_2_	CNT-ZnO	CNT-TiO_2_-ZnO	CNT-ZnO-TiO_2_
S_BET_/m^2^ g^−1^	94	54	75	43	31

**Table 5:** Results of the photocatalytic experiments.

Samples	Decomposition	k_app_	R^2^
	%	10^−4^ min^−1^	-
Photolysis	1.7	0.9	0.9485
P25 TiO_2_	22.4	10.6	0.9989
CNT-TiO_2_	19.9	10.8	0.9355
CNT-ZnO	41.5	26.0	0.9759
CNT-TiO_2_-ZnO	27.8	16.3	0.9592
CNT-ZnO-TiO_2_	11.1	6.4	0.8844

## Supplementary Materials

The following are available online at https://www.mdpi.com/2079-4991/10/2/252/s1: Instruments used for the characterization; UV–Vis spectrum of the lamps used; EDX and XPS spectra of the samples; setup used for the gas sensing experiments.
