# Supplementary material for: Photocatalytic and Gas Sensitive Multiwalled Carbon Nanotube/TiO2-ZnO and ZnO-TiO2 Composites Prepared by Atomic Layer Deposition
Source: Nanomaterials (Basel). 2020 Jan 31;10(2):252. doi: 10.3390/nano10020252 (PMC7075193; doi:10.3390/nano10020252)
Supplement: Supplementary file 1 [file nanomaterials-10-00252-s001.pdf]

Supporting information for the manuscript

Photocatalytic and gas sensitive multiwalled carbon nanotube/TiO_2-ZnO and ZnO-TiO_2 composites prepared by atomic layer deposition

László Péter Bakos [1,*], Nóra Justh [1], Ulisses Carlo Moura da Silva Bezerra da Costa [1], Krisztina László [2], János László Lábár [3], Tamás Igricz [4], Katalin Varga–Josepovits [5], Pawel Pasierb [6], Elina Färm [7], Mikko Ritala [8], Markku Leskelä [8] and Imre Miklós Szilágyi [1]

1 Department of Inorganic and Analytical Chemistry, Budapest University of Technology and Economics, Szent Gellért tér 4., H-1111 Budapest, Hungary; justh.nora@gmail.com (N.J.); ulissesccosta@gmail.com (U.C.M.d.S.B.d.C.); imre.szilagyi@mail.bme.hu (I.M.S.)

2 Department of Physical Chemistry and Materials Science, Budapest University of Technology and Economics, P.O. Box 92, H-1521 Budapest, Hungary; klaszlo@mail.bme.hu

3 Institute for Technical Physics and Materials Science, Research Centre of Energy, Hungarian Academy of Sciences, Konkoly–Thege út 29–33., H-1121 Budapest, Hungary; labar.janos@energia.mta.hu

4 Department of Organic Chemistry and Technology, Budapest University of Technology and Economics, Budafoki út 8., H-1111 Budapest, Hungary; igricz.tamas@gmail.com

5 Department of Atomic Physics, Budapest University of Technology and Economics, Budafoki út 8., H-1111 Budapest, Hungary; flip@eik.bme.hu

6 Department of Inorganic Chemistry, AGH University of Science and Technology, Mickiewicza 30., 30-059 Kraków, Poland; ppasierb@agh.edu.pl

7 ASM Microchemistry Oy, Pietari Kalmin katu 1F2, FI-00560 Helsinki, Finland; elina.farm@helsinki.fi

8 Department of Chemistry, University of Helsinki, P.O. Box 55, FI-00014, Helsinki, Finland; mikko.ritala@helsinki.fi (M.R.); markku.leskela@helsinki.fi (M.L.)

* Correspondence: laszlobakos@hotmail.com

Hydroxil-functionalization of the nanotubes

According to the producer: "They are produced by Catalyzed Chemical Vapor Deposition and purified/functionalized using acid chemistry, KMnO4 in H2SO4 solution.". https://www.cheaptubes.com/product-category/functionalized-carbon-nanotubes/oh-functionalized-carbon-nanotubes/)

Characterization parameters

TG/DTA measurements were conducted on a TA Instruments SDT 2960 simultaneous TG/DTA device in nitrogen and air atmospheres (130 cm^3 min^{-1}) using an open platinum crucible and 10 °C min^{-1} heating rate.

Raman spectra were collected by on a Jobin Yvon Labram Raman instrument equipped with an Olympus BX41 microscope using a frequency duplicated green Nd-YAG laser with 532 nm wavelength.

Powder XRD patterns were recorded on a PANanalytical X'Pert Pro MPD X-ray diffractometer with Cu Kα radiation.

SEM images were taken by a Hitachi S-4800 FESEM equipment. EDX spectra were measured by a JEOL JSM-5500LV scanning electron microscope equipped with an iXRF EDX device at 20 kV. Average EDX data were calculated from three measurements on each sample.

TEM-EELS-SAED images were recorded with a JEOL 3010 transmission electron microscope operating at 300 keV. Prior to the measurements, the samples were dispersed in EtOH, dropped on Cu grids covered with a Quantifoil Holey Carbon film and dried. Nanotubes protruding over the holes were examined to eliminate the effect of supporting film. The EELS elemental maps (in EFTEM mode) were recorded with a GATAN Tridiem imaging filter. Electron diffraction (SAED)-like patterns were obtained by Fourier-transforming (FFT) the TEM images.

X–ray Photoelectron (XPS) spectra were recorded by a VG Microtech instrument consisting of a XR3E2 X–ray source, a twin anode (Mg K and Al K) and a CLAM 2 hemispherical analyzer using Mg K radiation. Detailed scans were recorded with 50 eV pass energy at (0.05 eV/1.5 s). The spectrometer was calibrated with the binding energy of the C1s line (285 eV).

Nitrogen adsorption/desorption isotherms were measured at -196 °C with a Nova2000e (Quantachrome) computer controlled apparatus. The apparent surface area (S_{BET}) was calculated using the Brunauer-Emmett-Teller (BET) model.

The photocatalytic activity was investigated by placing 1.0 mg sample into 3 mL aqueous solution of methyl orange dye (initial concentration: $4\cdot10^{-5}$ M) into quartz cuvettes. For reference, P25 Degussa TiO_2 was used. After one hour in dark to reach the adsorption equilibrium, the cuvettes were placed between two parallel Osram 18 W blacklight lamps (see their spectra on Fig. S1), 5 cm from both. The decomposition of the methyl orange was followed for four hours by measuring every 30 min the absorption of its 464 nm peak by a a Jasco V-550 UV-Vis spectrophotometer. To measure the gas sensitivity, Al_2O_3 sensor sheets with interdigitated Pt electrodes were used, onto which Pt wires were contacted with Au suspension and dried at 120 °C for 30 min. Sensing layers were produced by drop coating the terpenol slurry of the powder samples onto the sensor sheets. NH_3 sensing properties were tested at different concentrations (500, 1000, 2500, 5000 ppm in nitrogen) at 25 and 150 °C. The setup is shown on Fig. S27-28.

UV-Vis spectrum

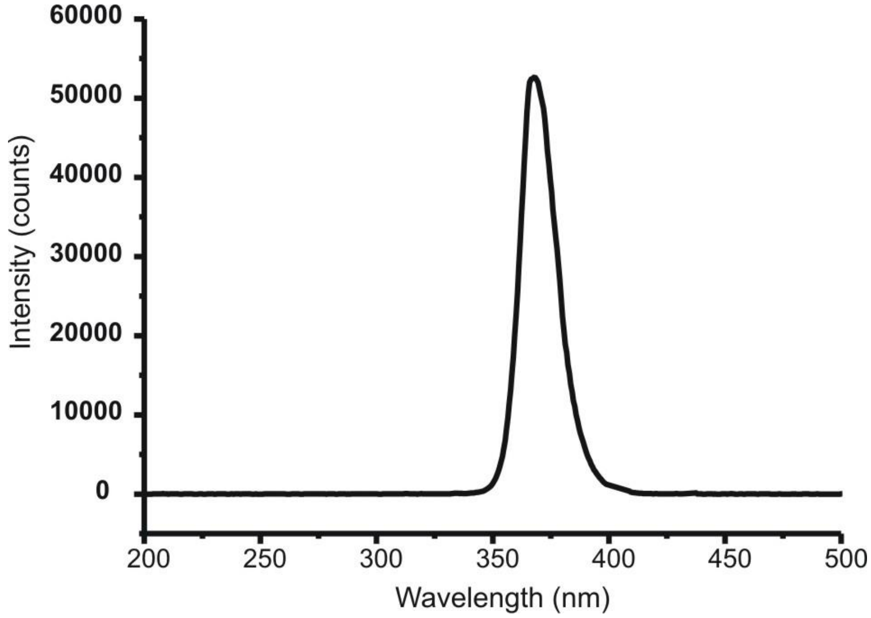

Fig. S1. UV-Vis spectrum of the Osram 18 W blacklight lamps used for photocatalysis reactions

EDX spectra

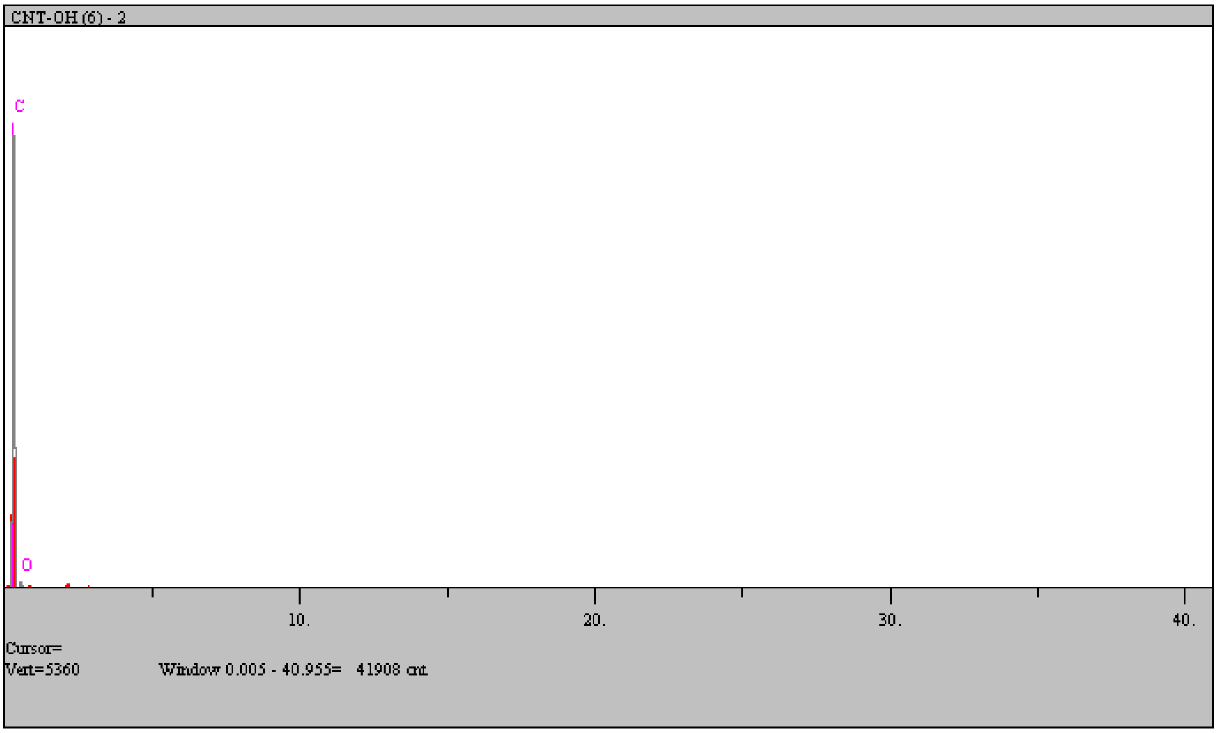

Figure S2. EDX spectrum for CNT-OH

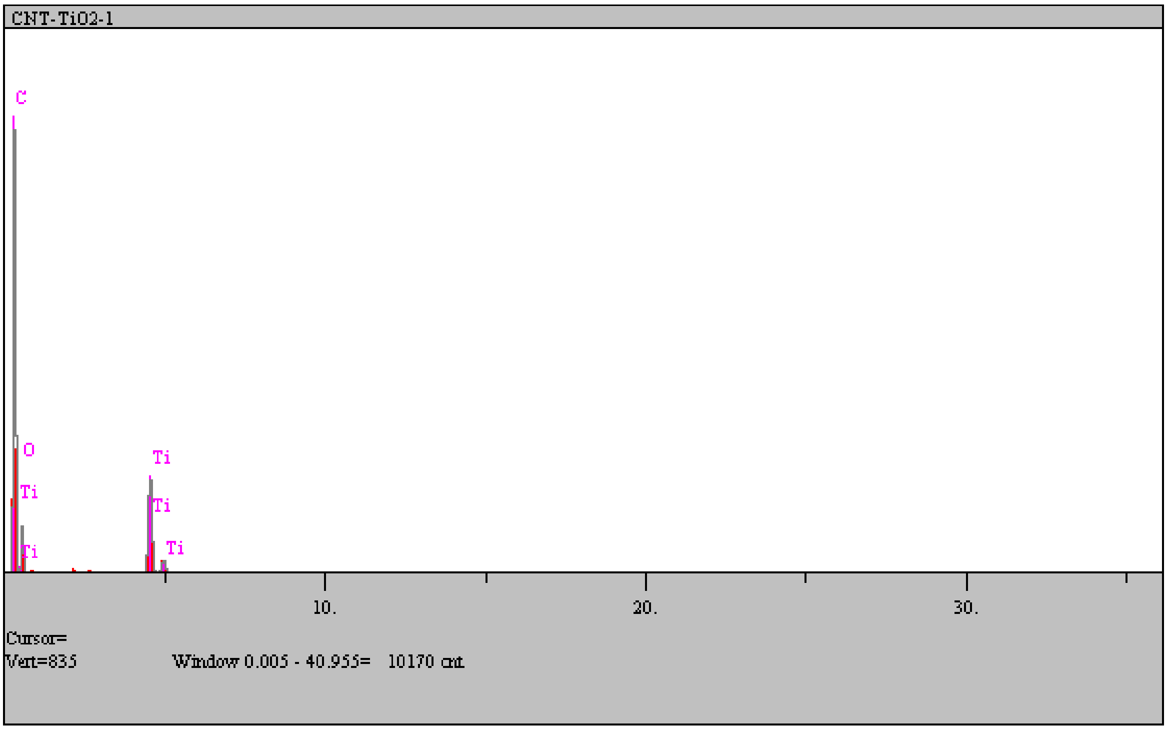

Figure S3. EDX spectrum for CNT-TiO_2

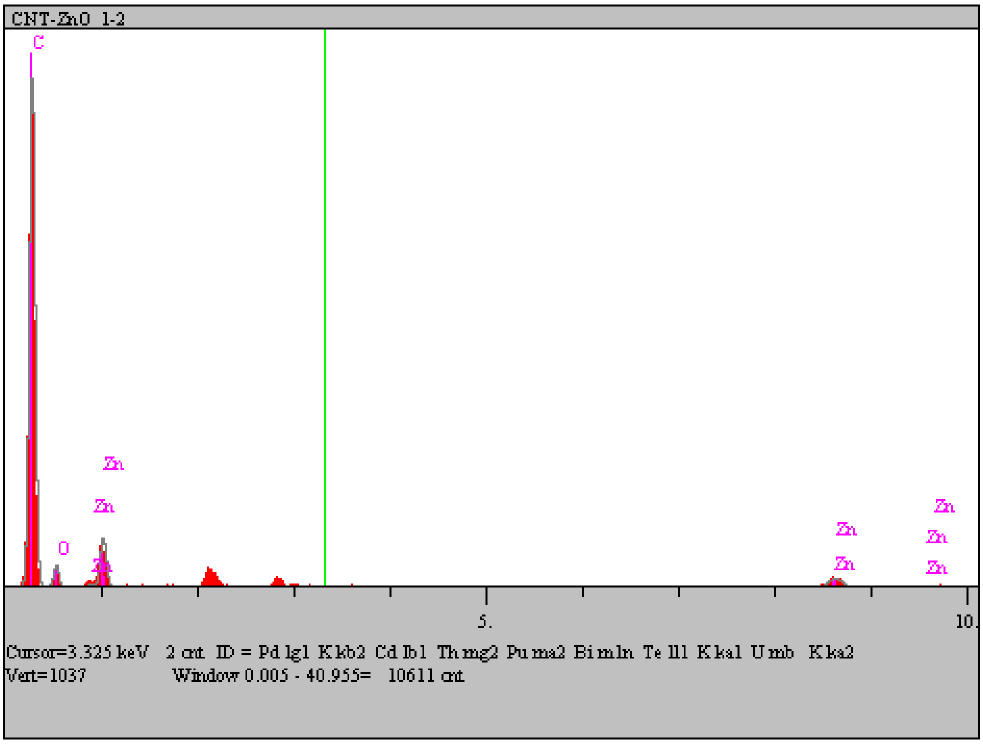

Figure S4. EDX spectrum for CNT-ZnO

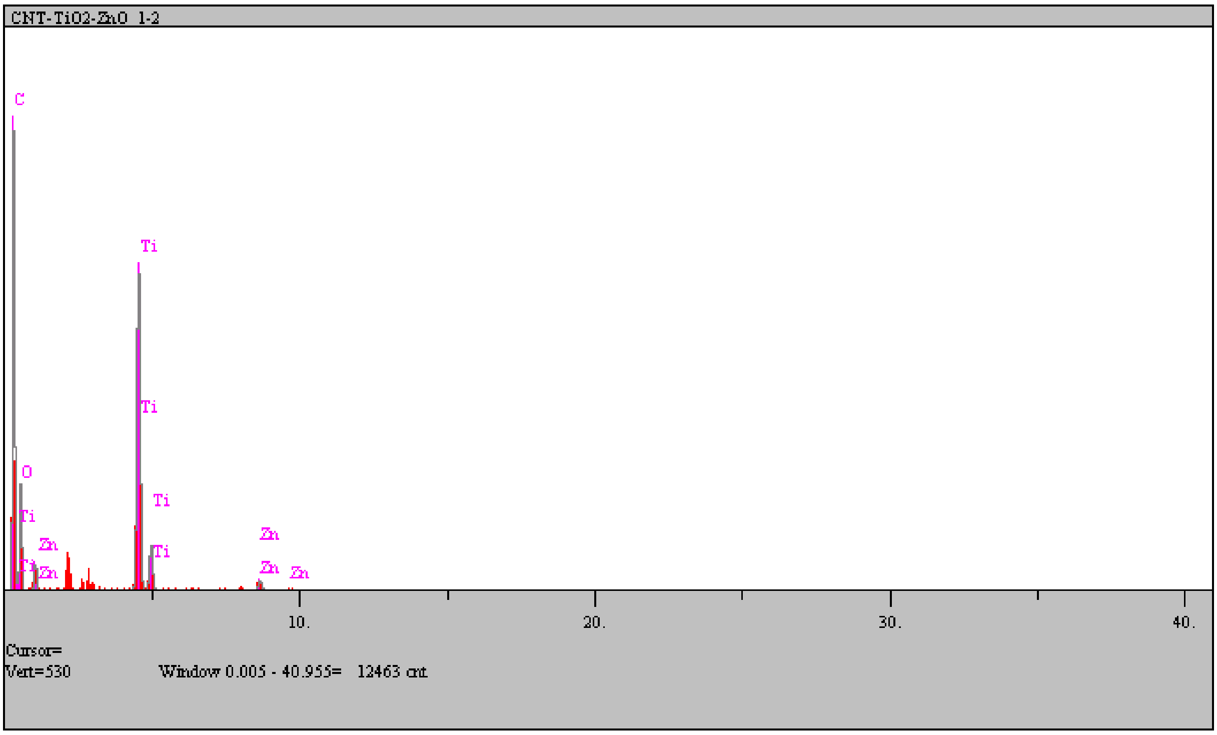

Figure S5. EDX spectrum for CNT-TiO_2-ZnO

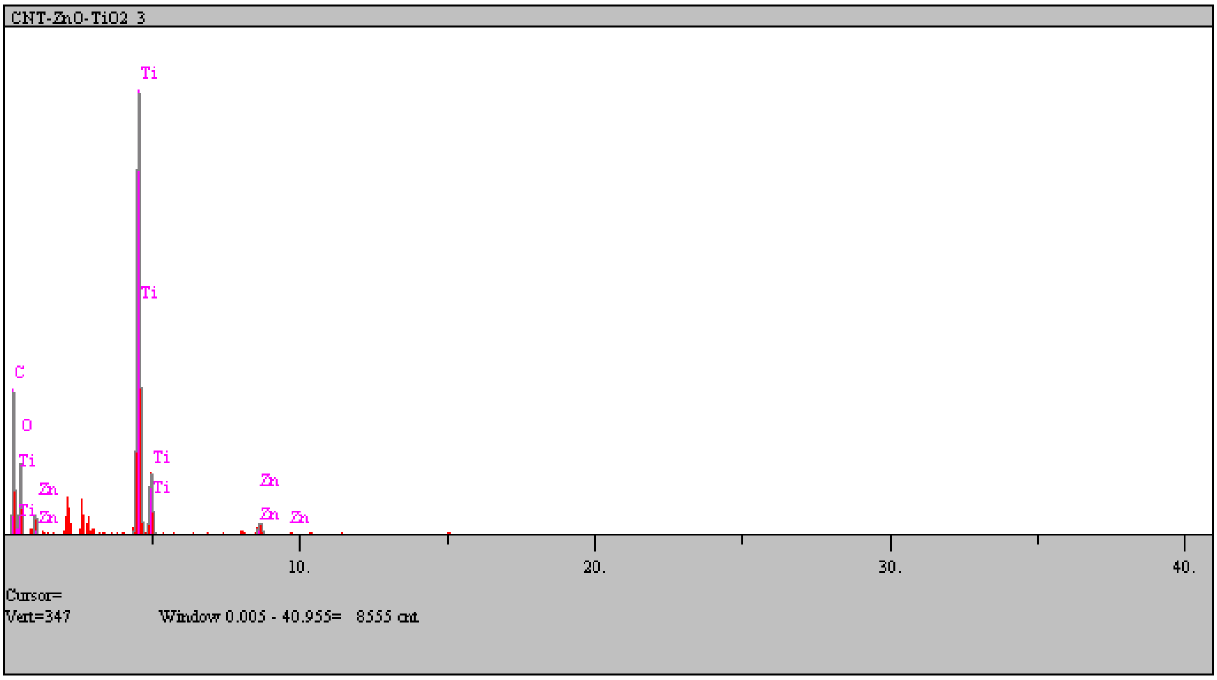

Figure S6. EDX spectra for CNT-ZnO-TiO_2

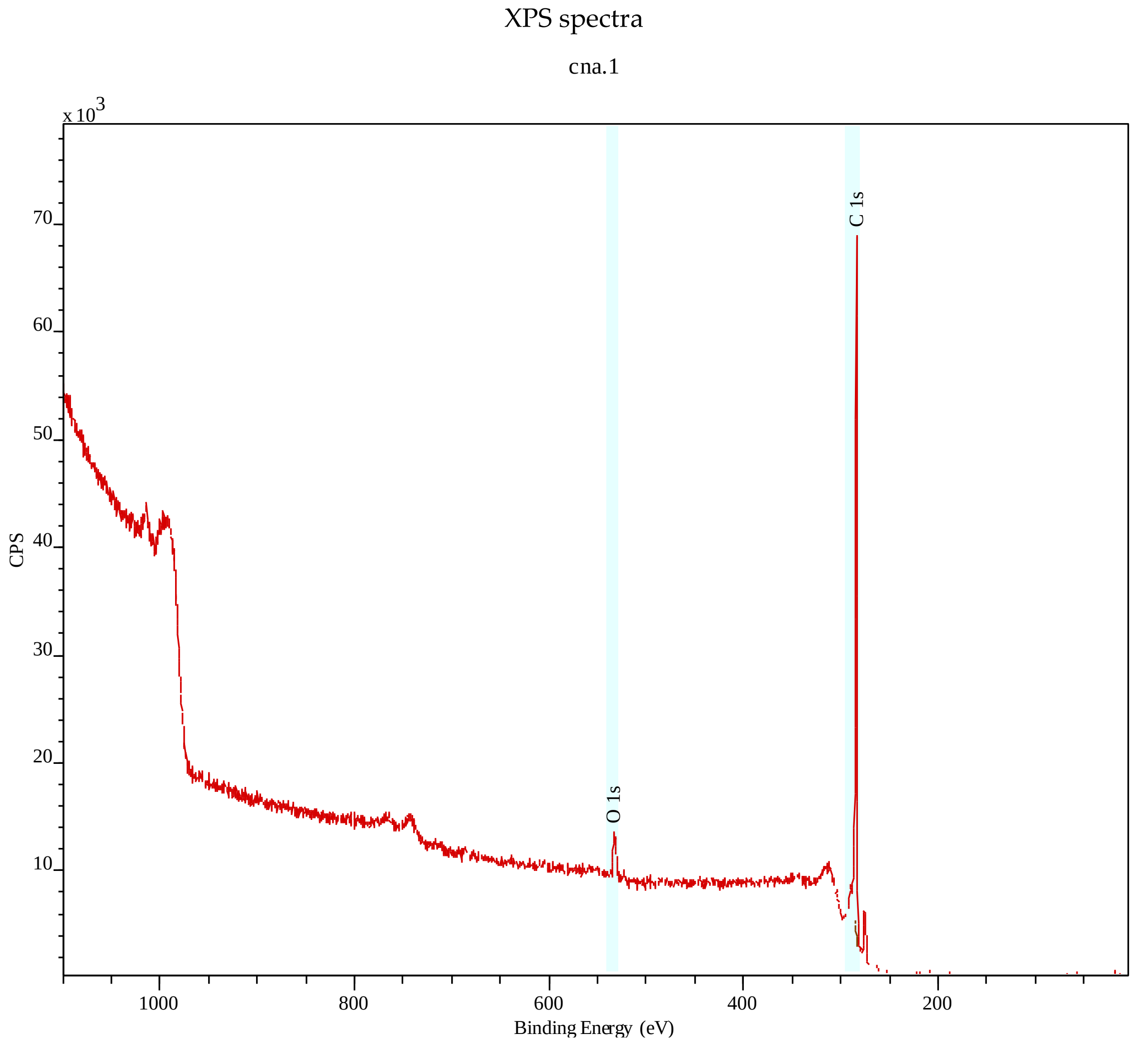

Figure S7. XPS spectrum for CNT-OH

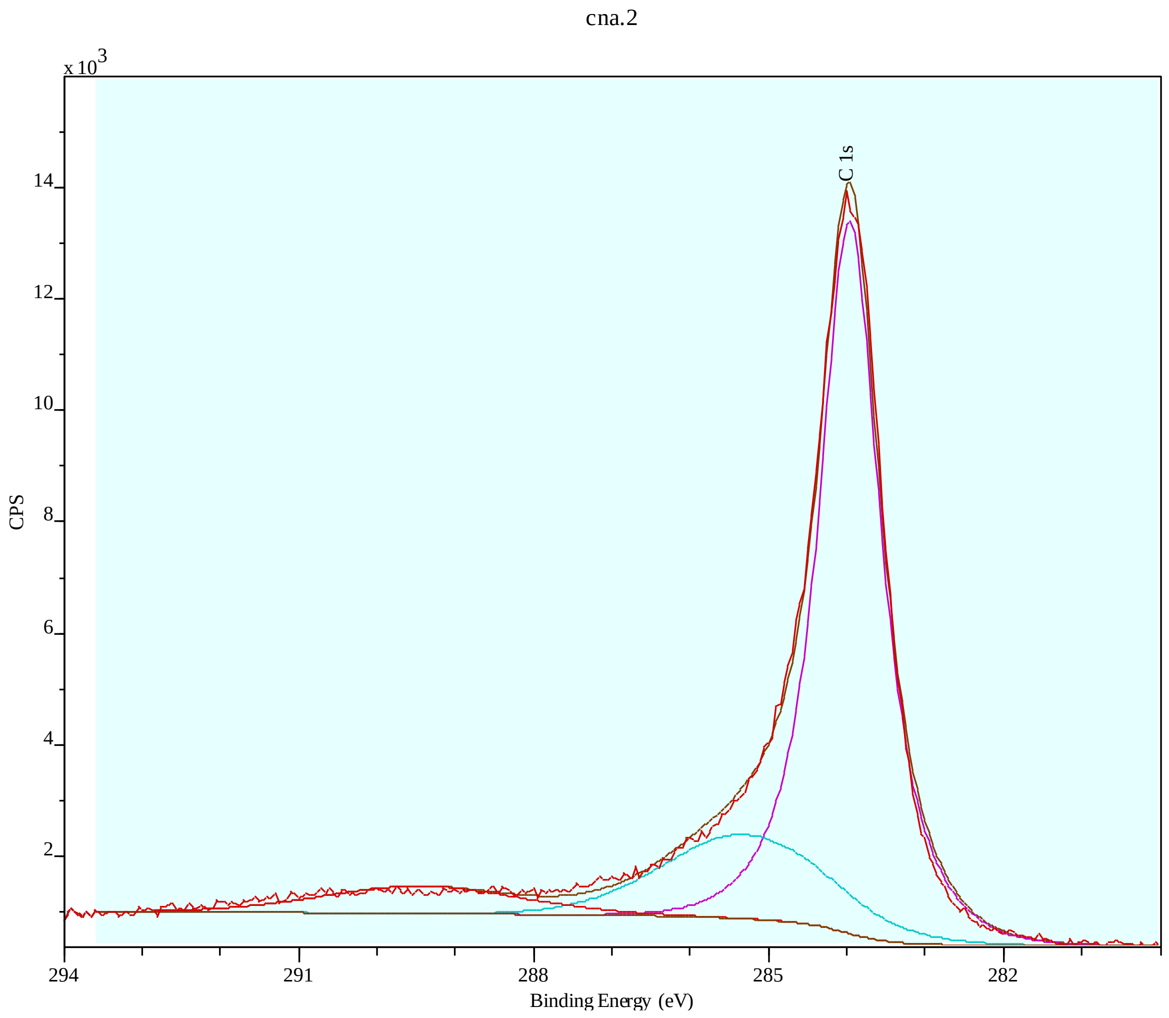

Figure S8. Deconvolution of the C1s peak for CNT-OH

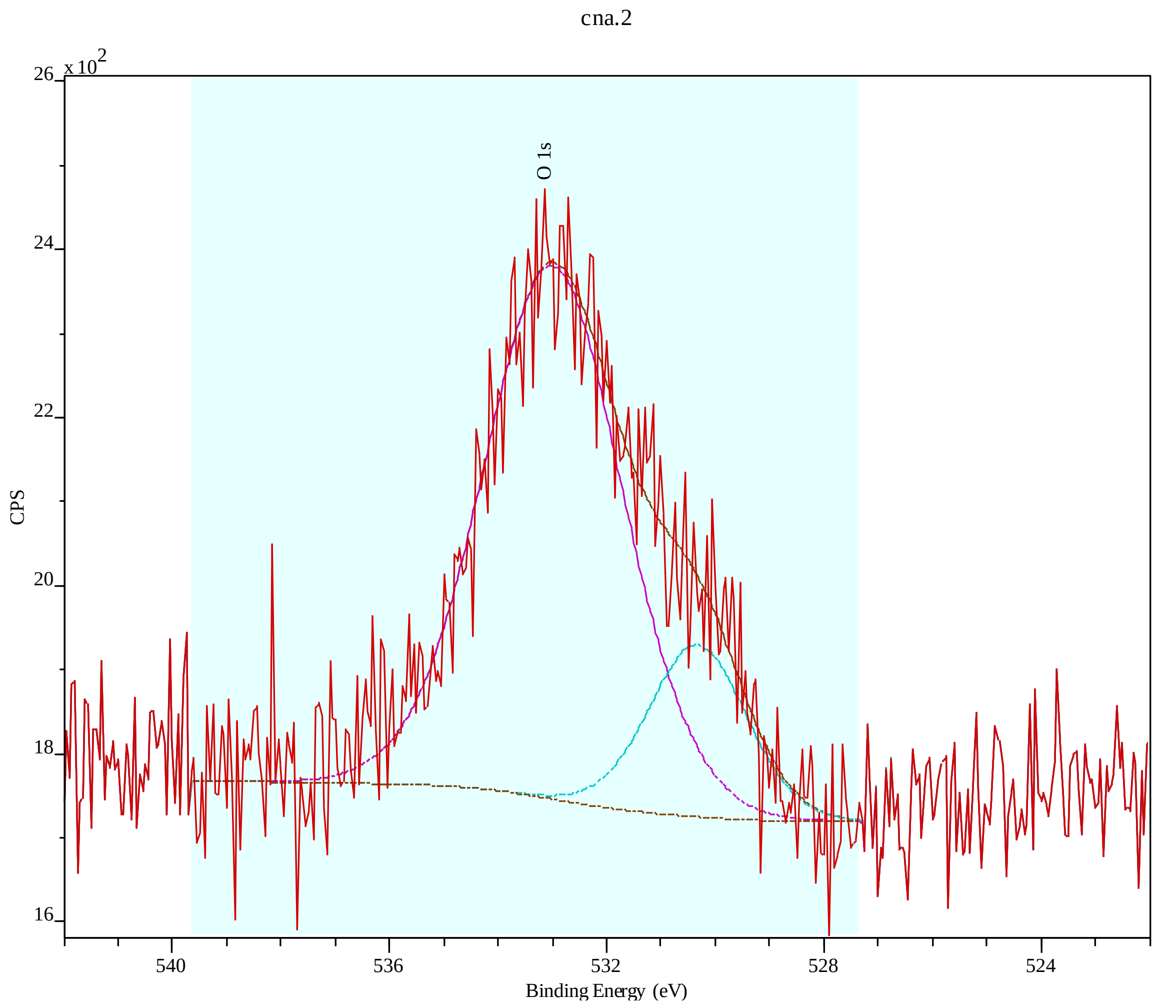

Figure S9. Deconvolution of the O1s peak for CNT-OH

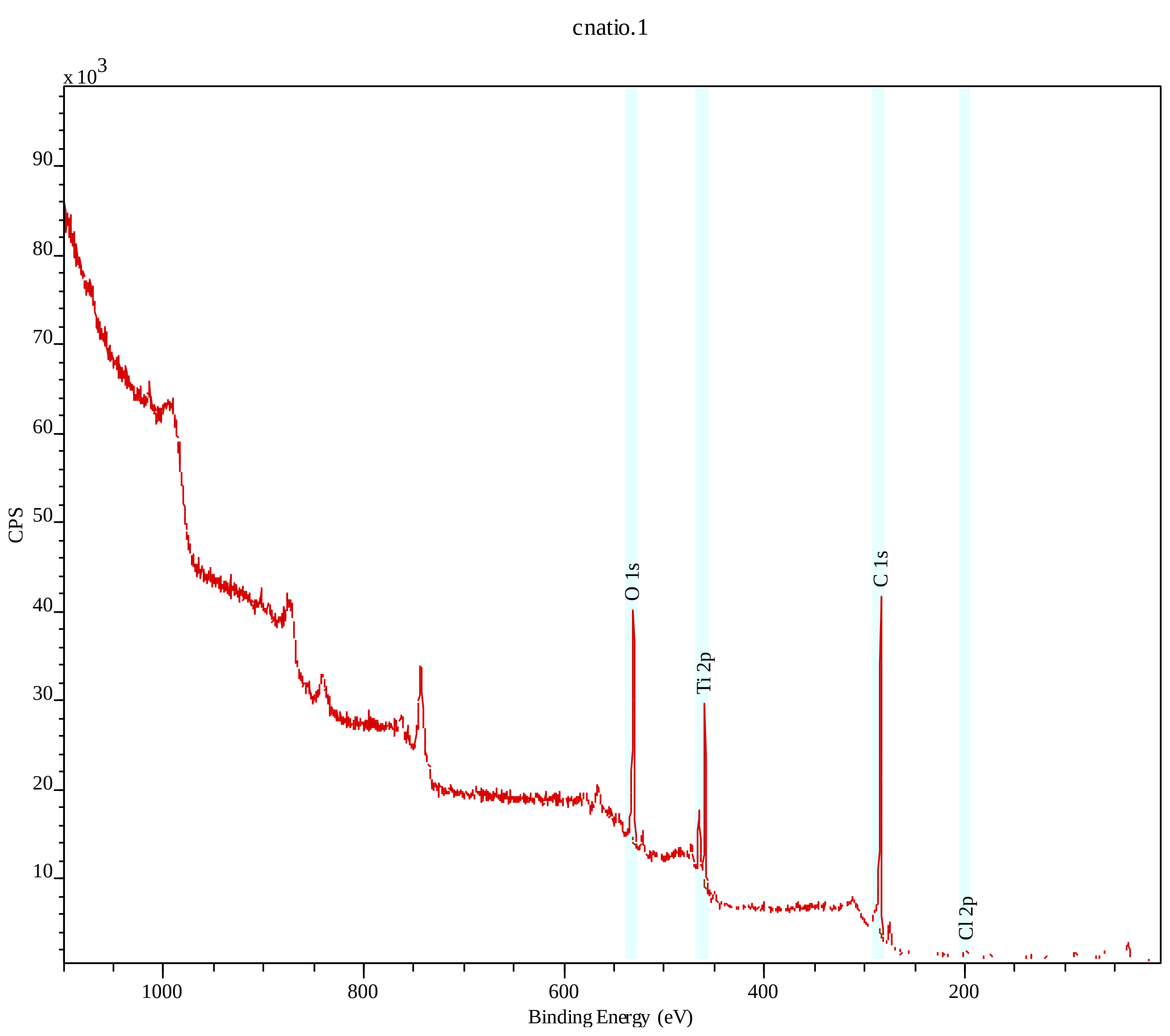

Figure S10. XPS spectrum for CNT-TiO_2

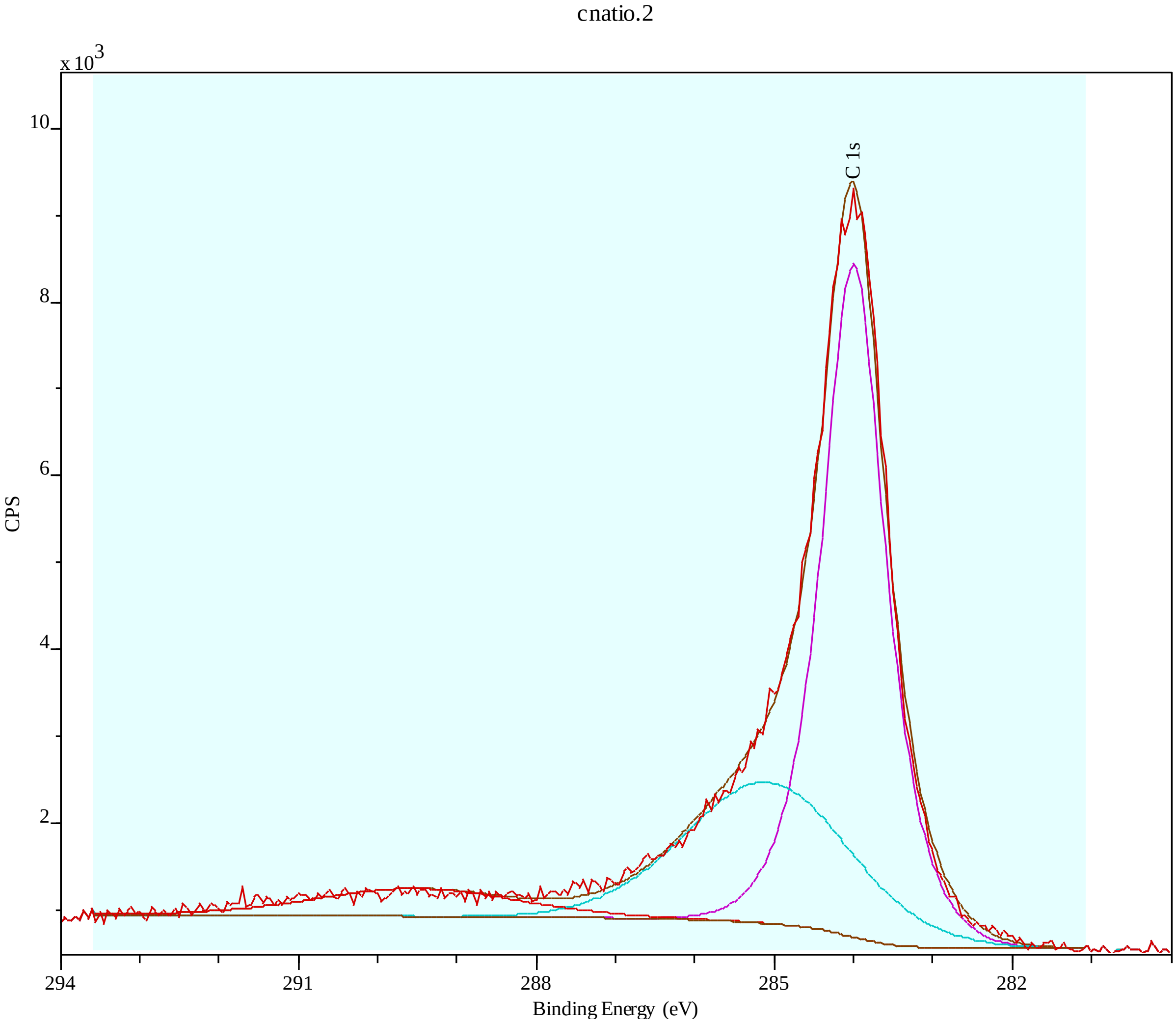

Figure S11. Deconvolution of the C1s peak for CNT-TiO_2

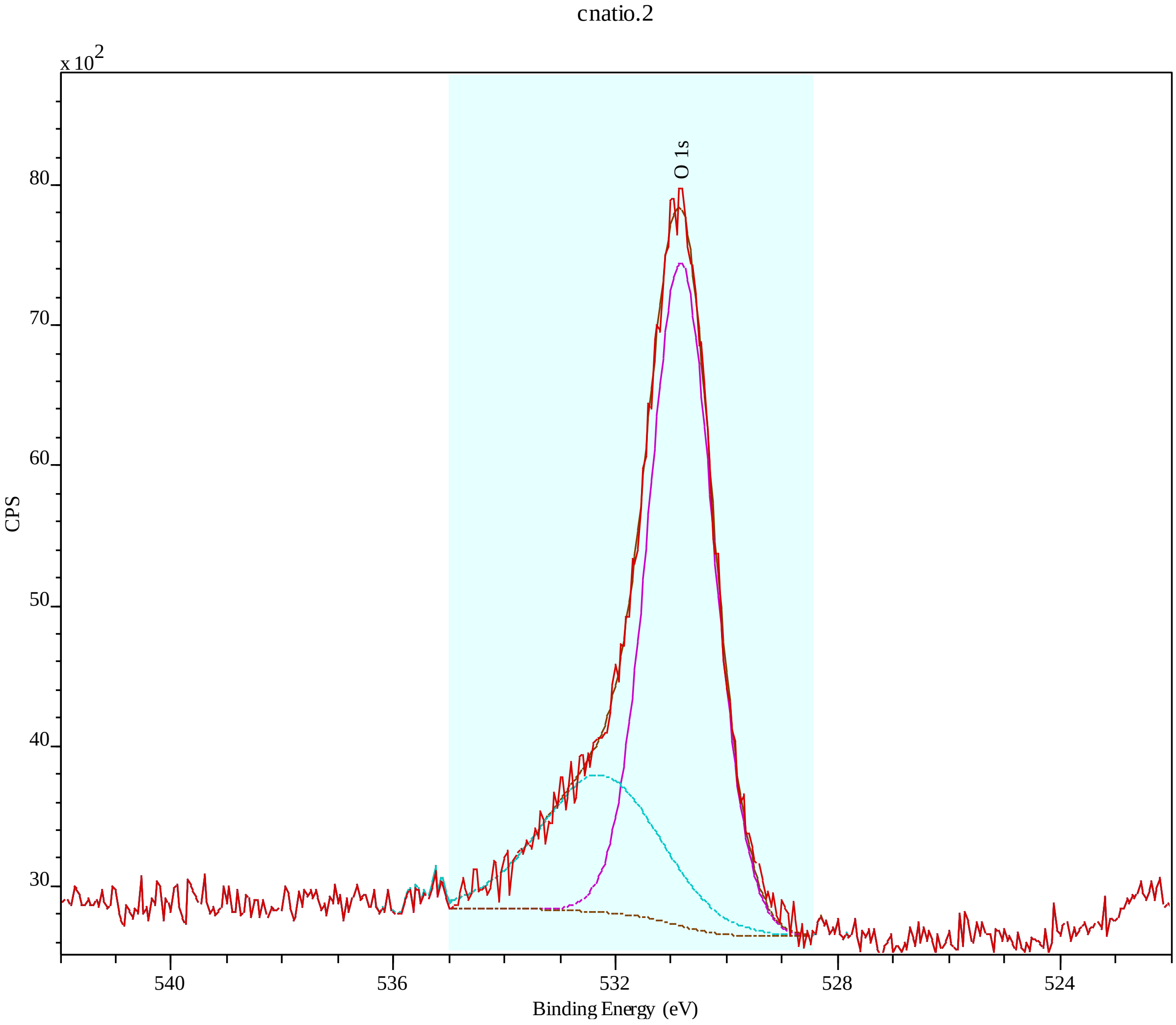

Figure S12. Deconvolution of the O1s peak for CNT-TiO_2

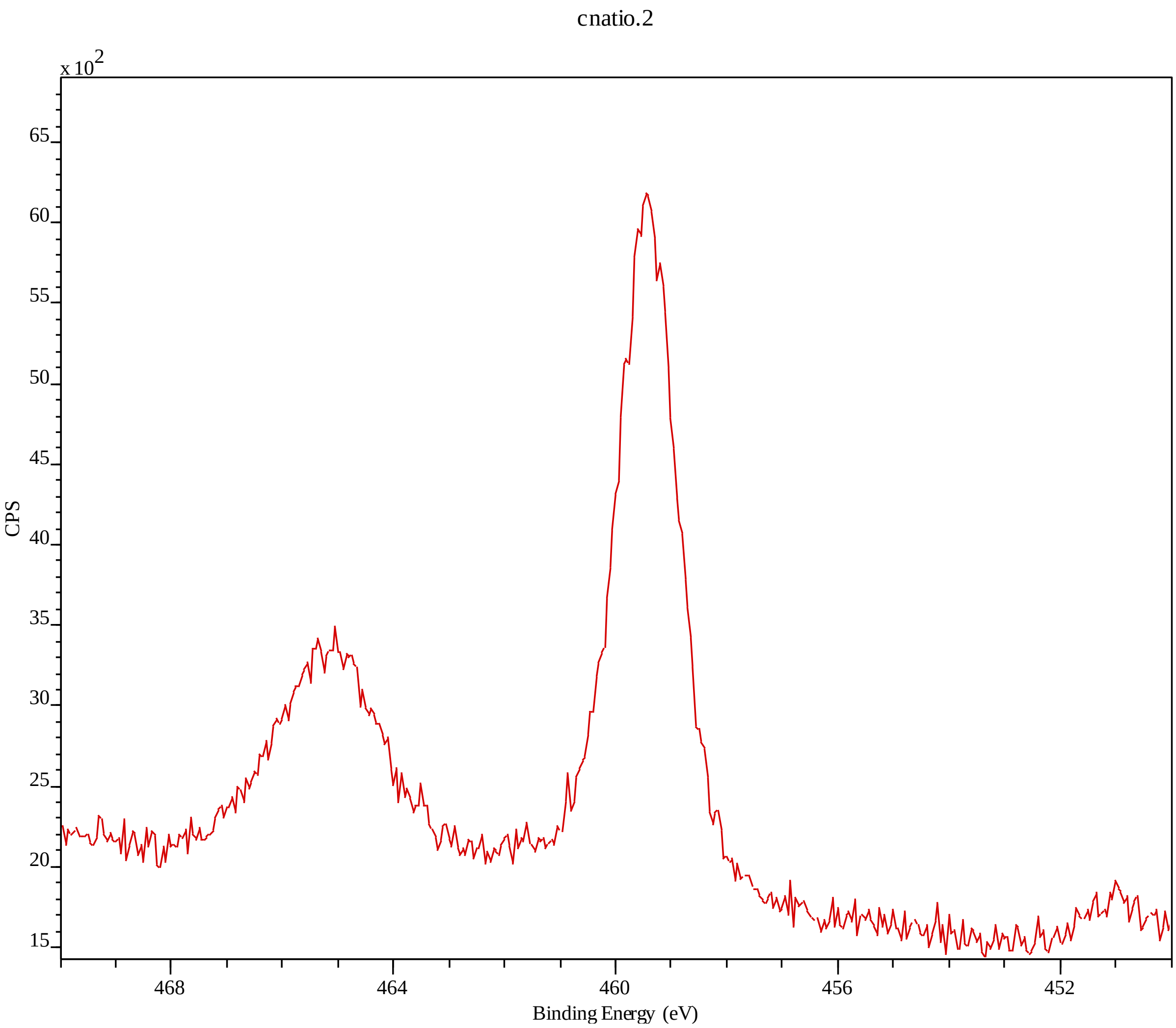

Figure S13. Ti2p peak for CNT- TiO_2

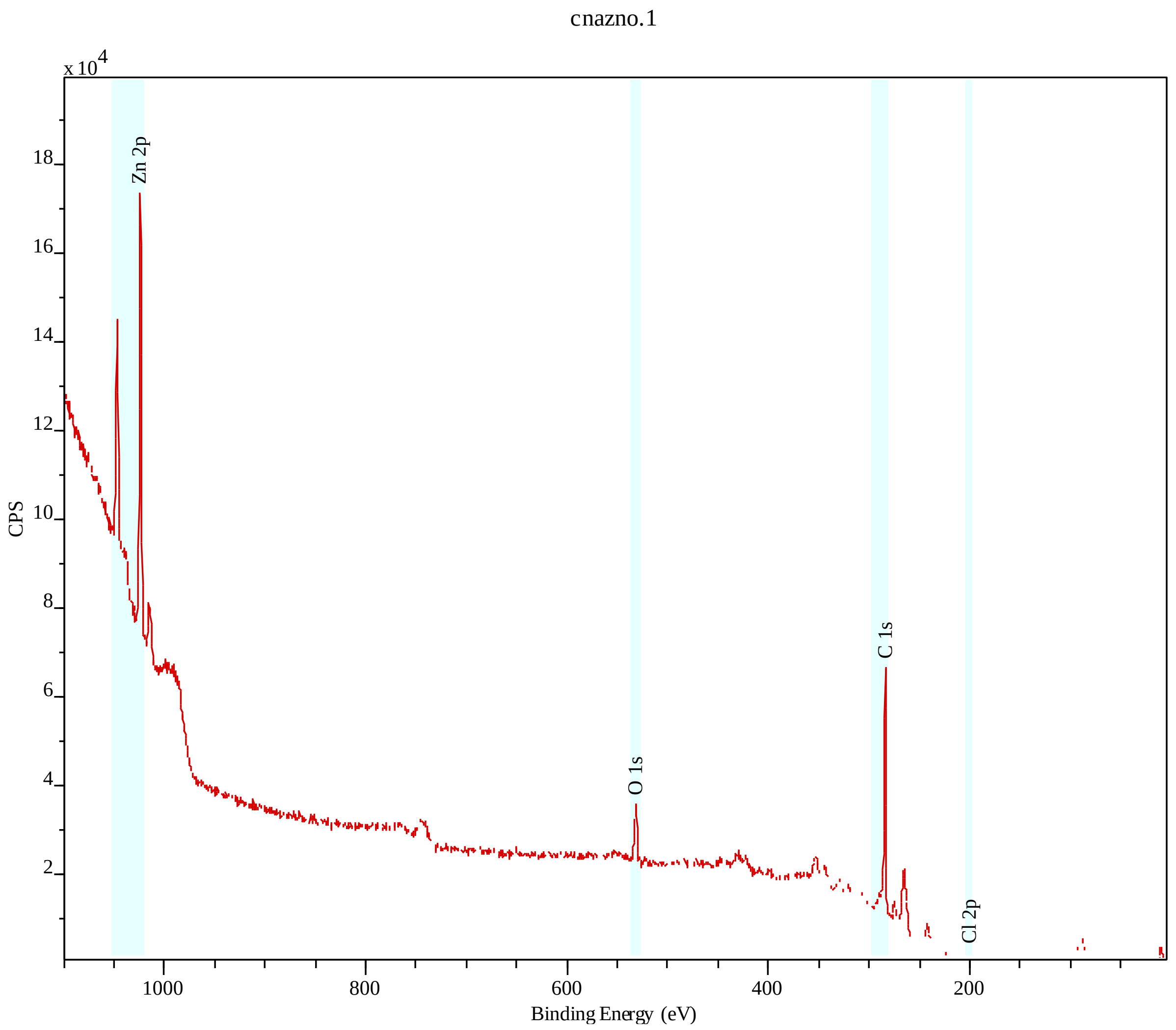

Figure S14. XPS spectrum for CNT-ZnO

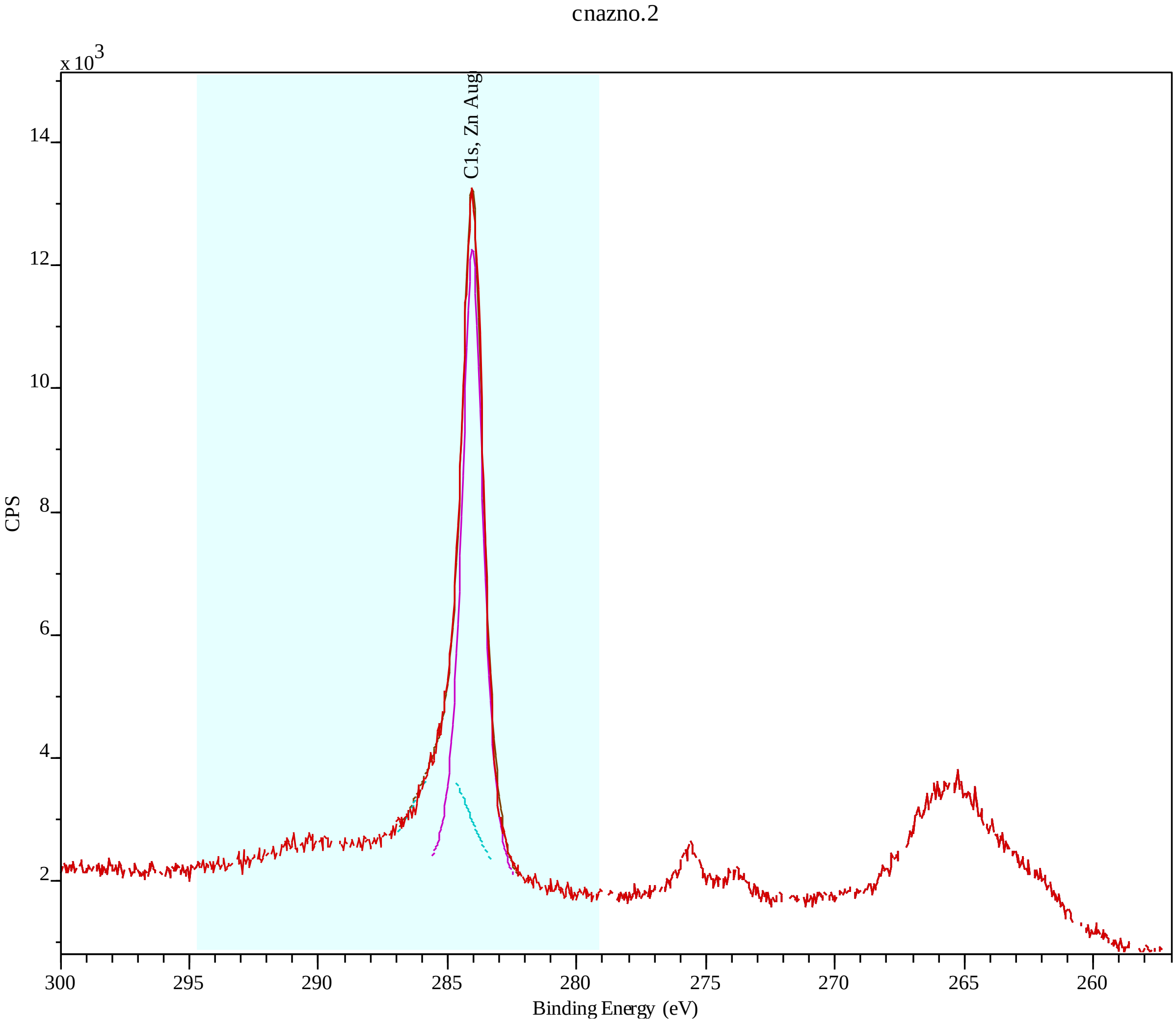

Figure S15. Deconvolution of the C1s peak for CNT-ZnO

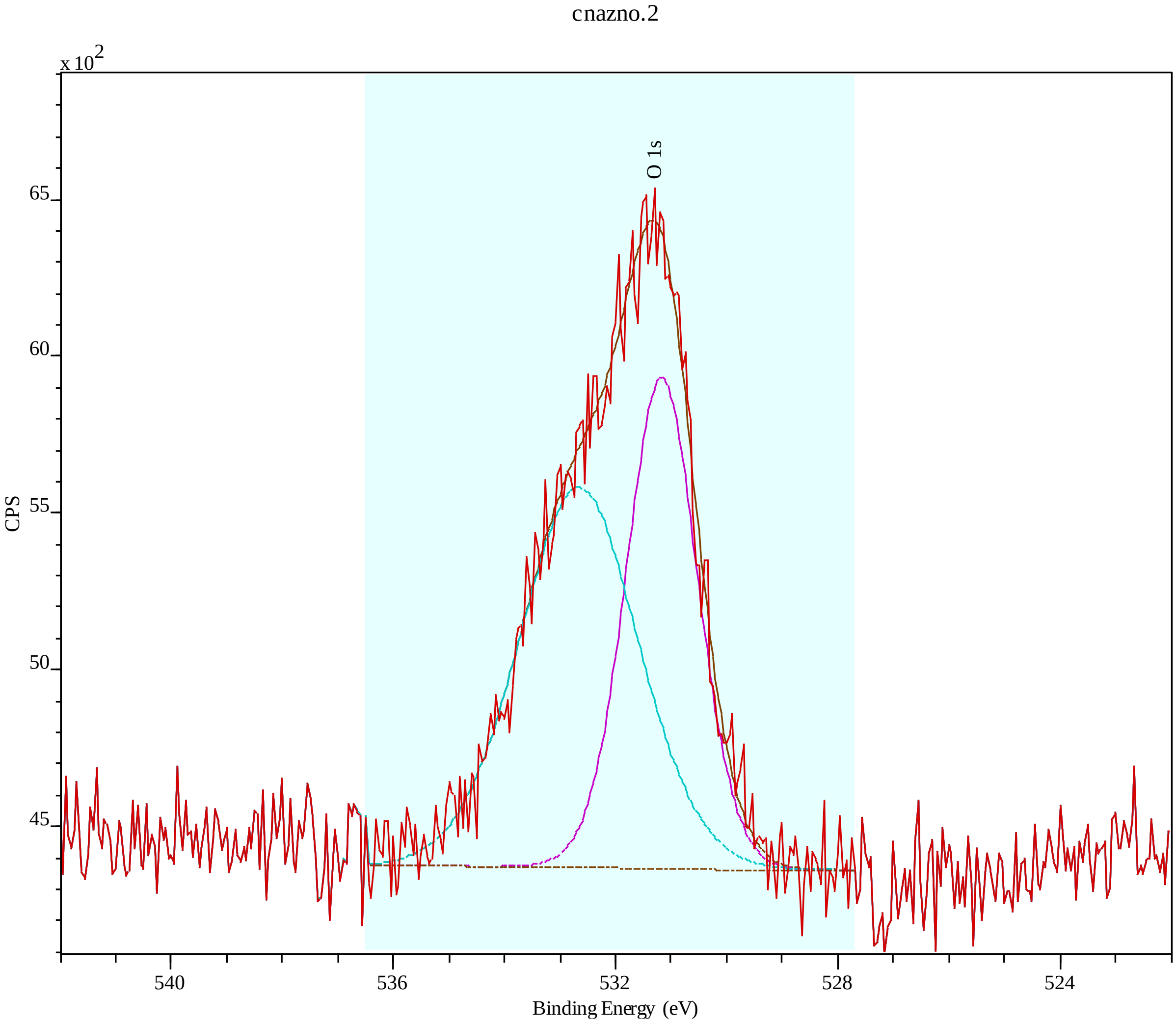

Figure S16. Deconvolution of the O1s peak for CNT-ZnO

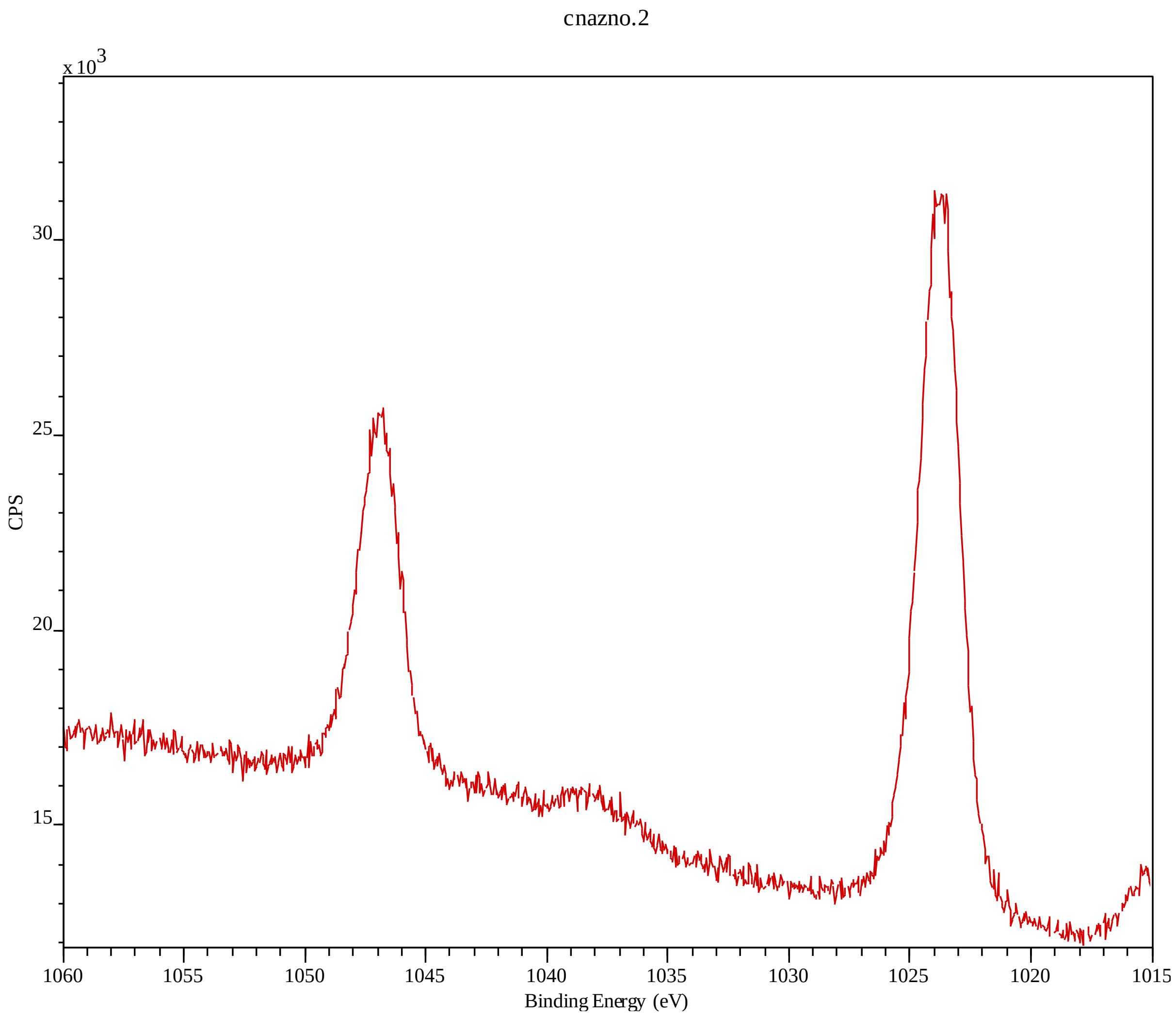

Figure S17. Zn2p peak for CNT-ZnO

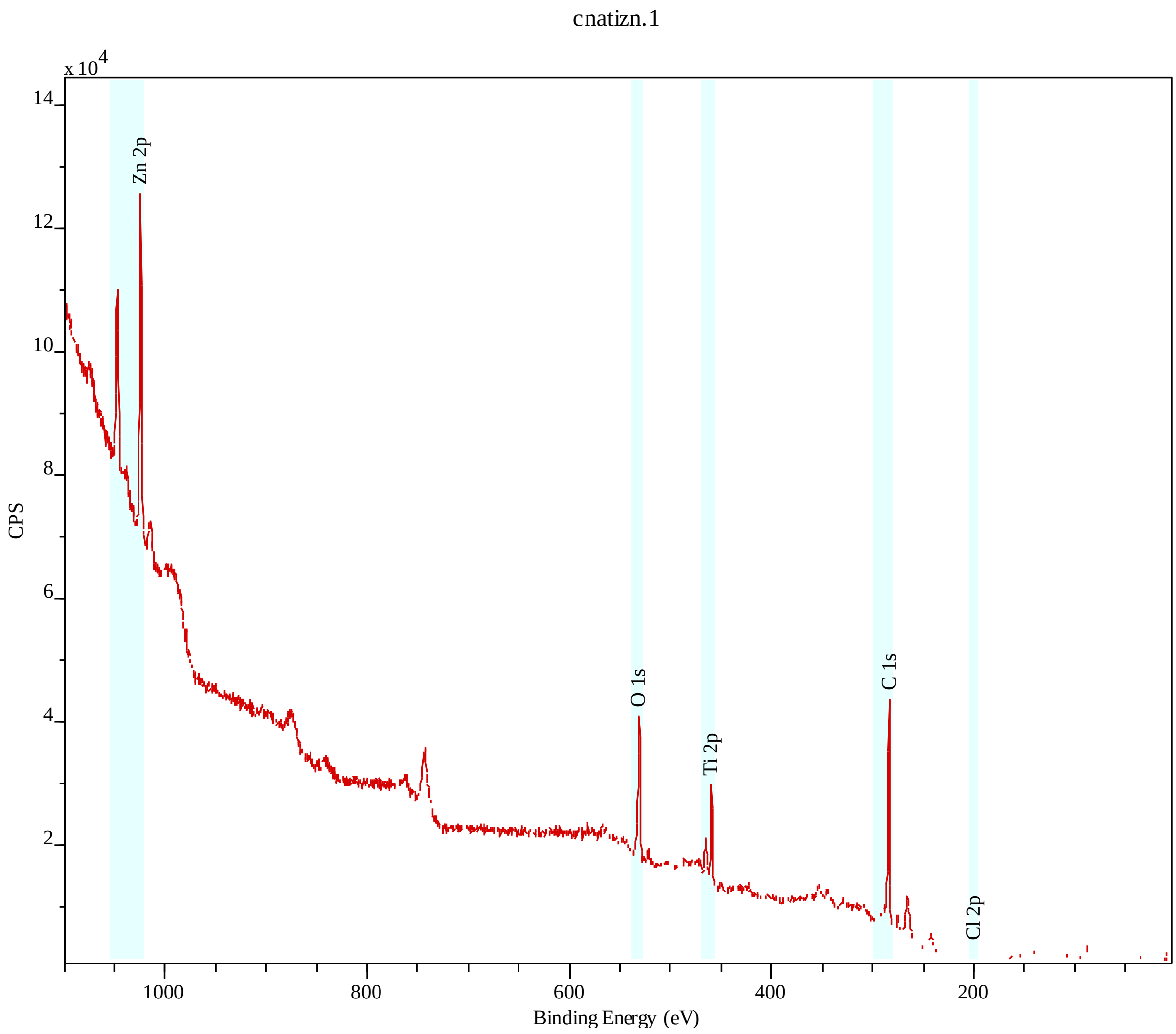

Figure S18. XPS spectrum for CNT-TiO_2-ZnO

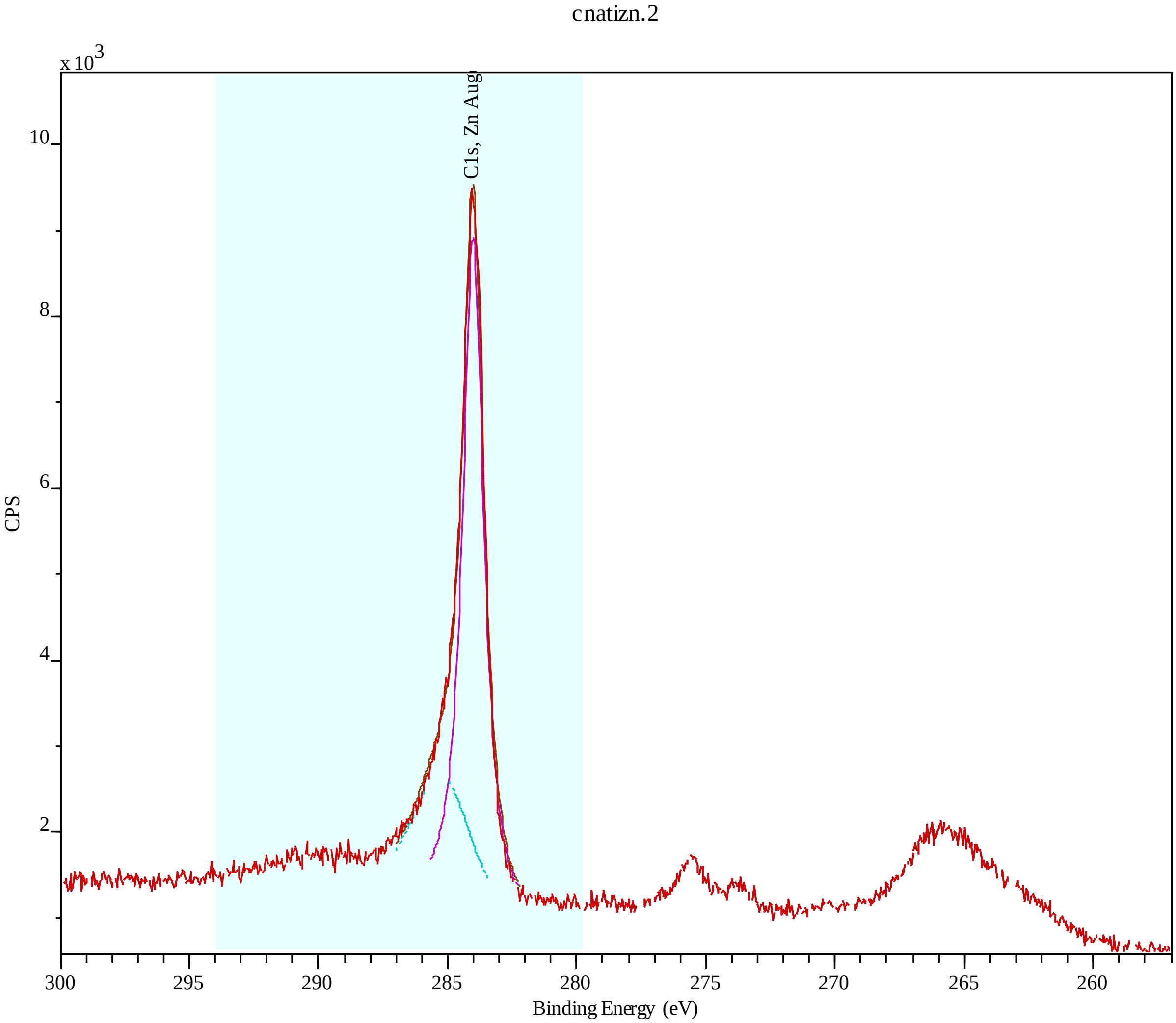

Figure S19. Deconvolution of the C1s peak for CNT-TiO_2-ZnO

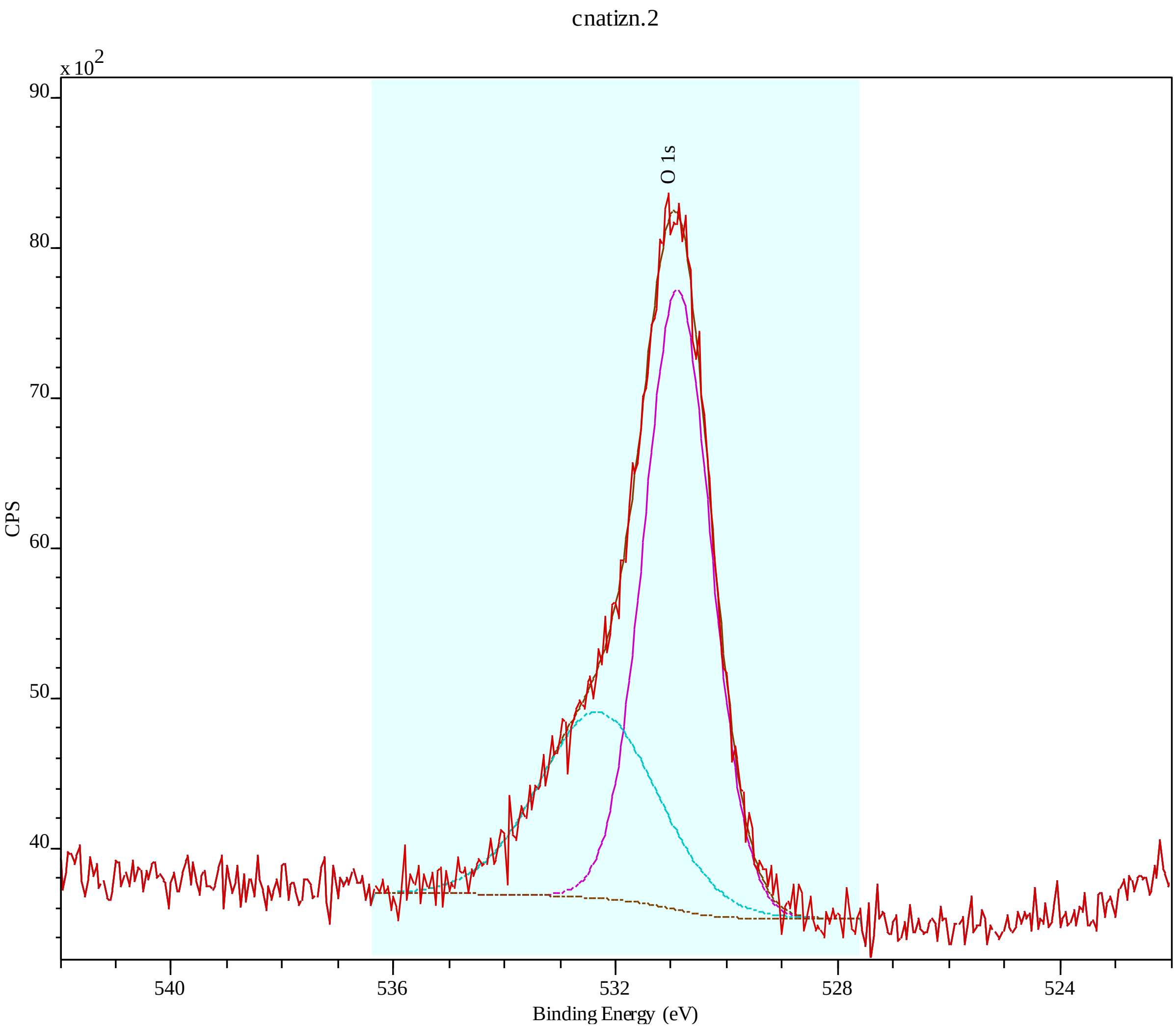

Figure S20. Deconvolution of the O1s peak for CNT-TiO_2-ZnO

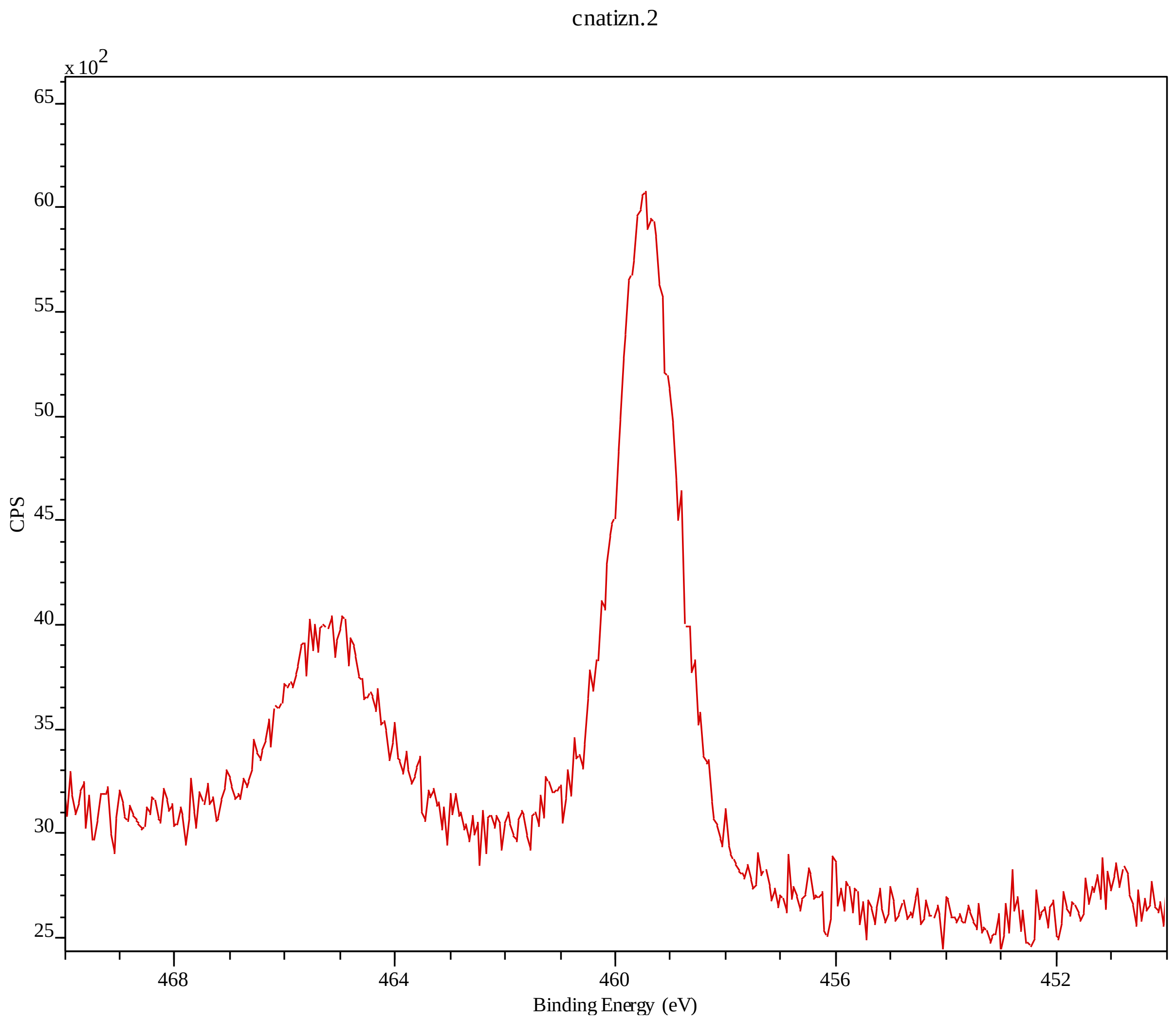

Figure S21. Ti2p peak for CNT-TiO_2-ZnO

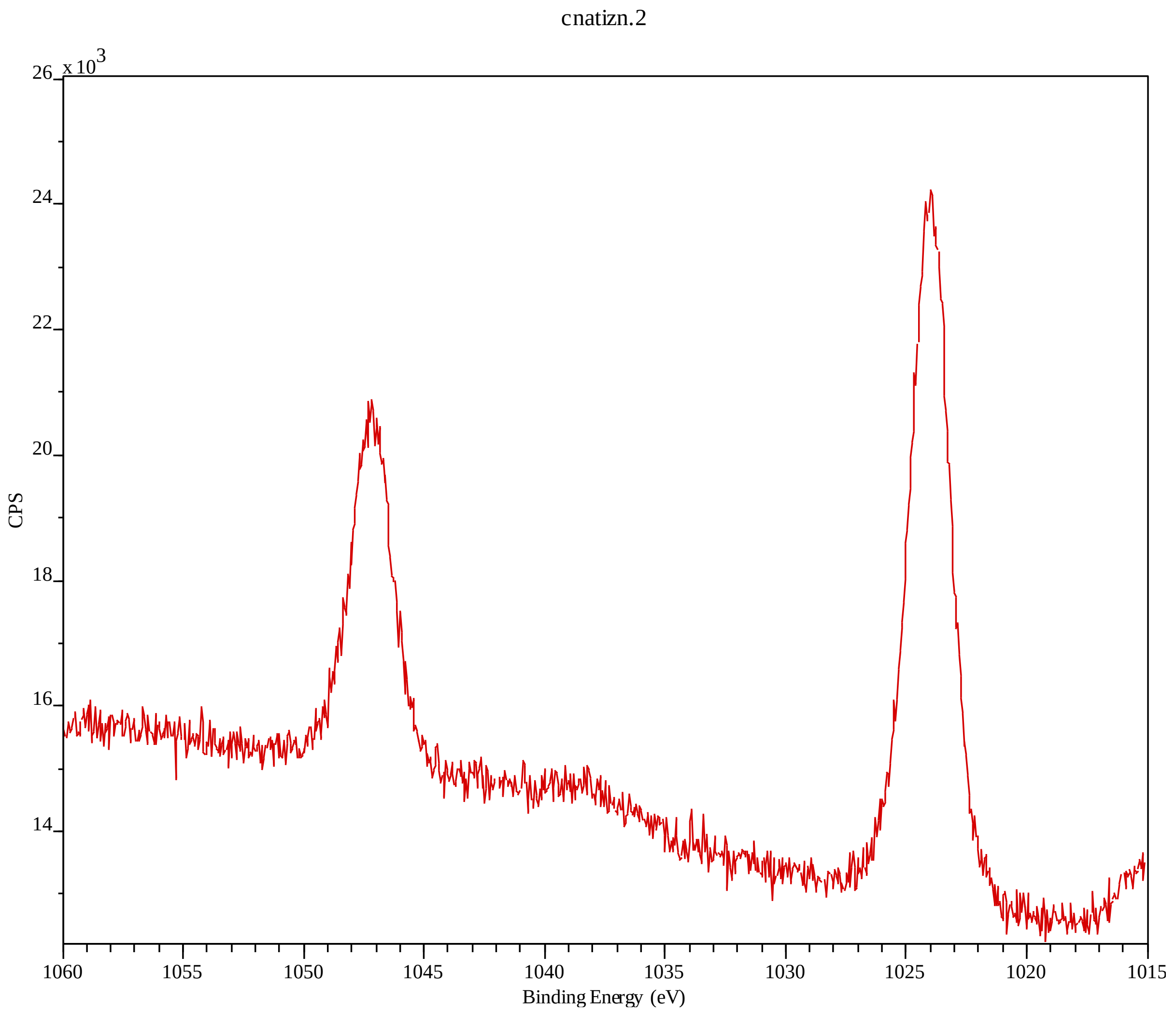

Figure S22. Zn2p peak for CNT-TiO_2-ZnO

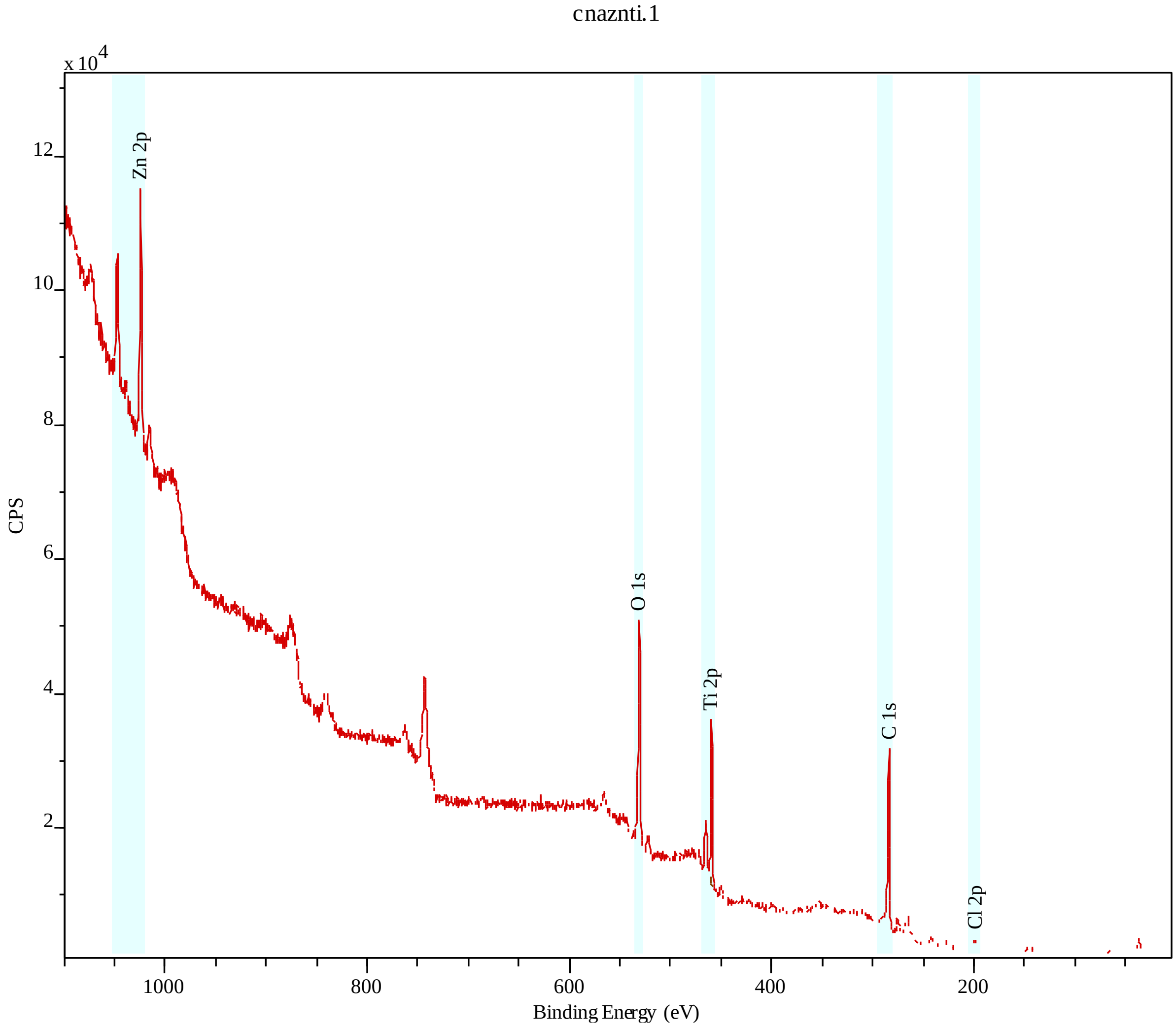

Figure S23. XPS spectrum for CNT-ZnO-TiO_2

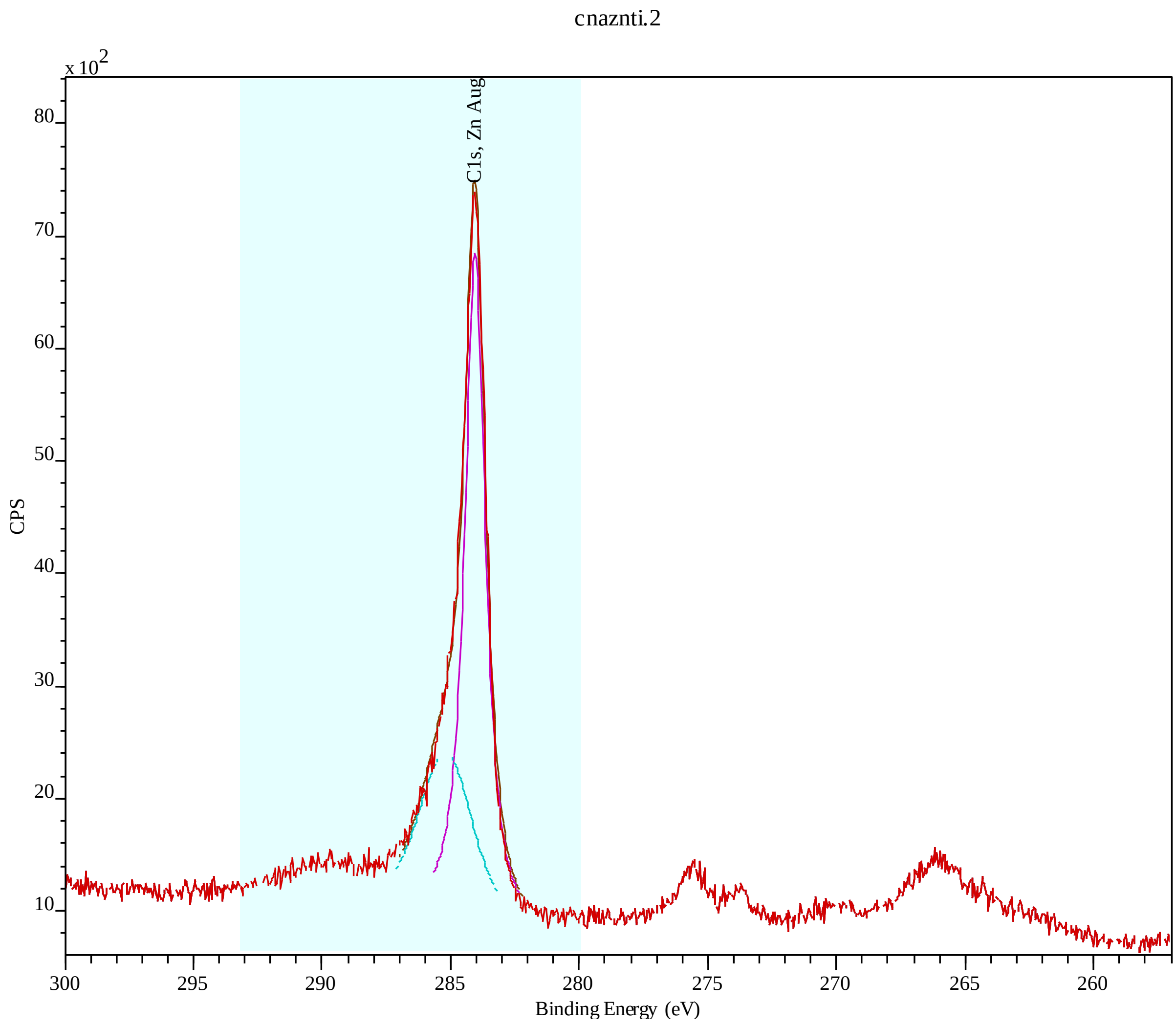

Figure S24. Deconvolution of the C1s peak for CNT-ZnO-TiO_2

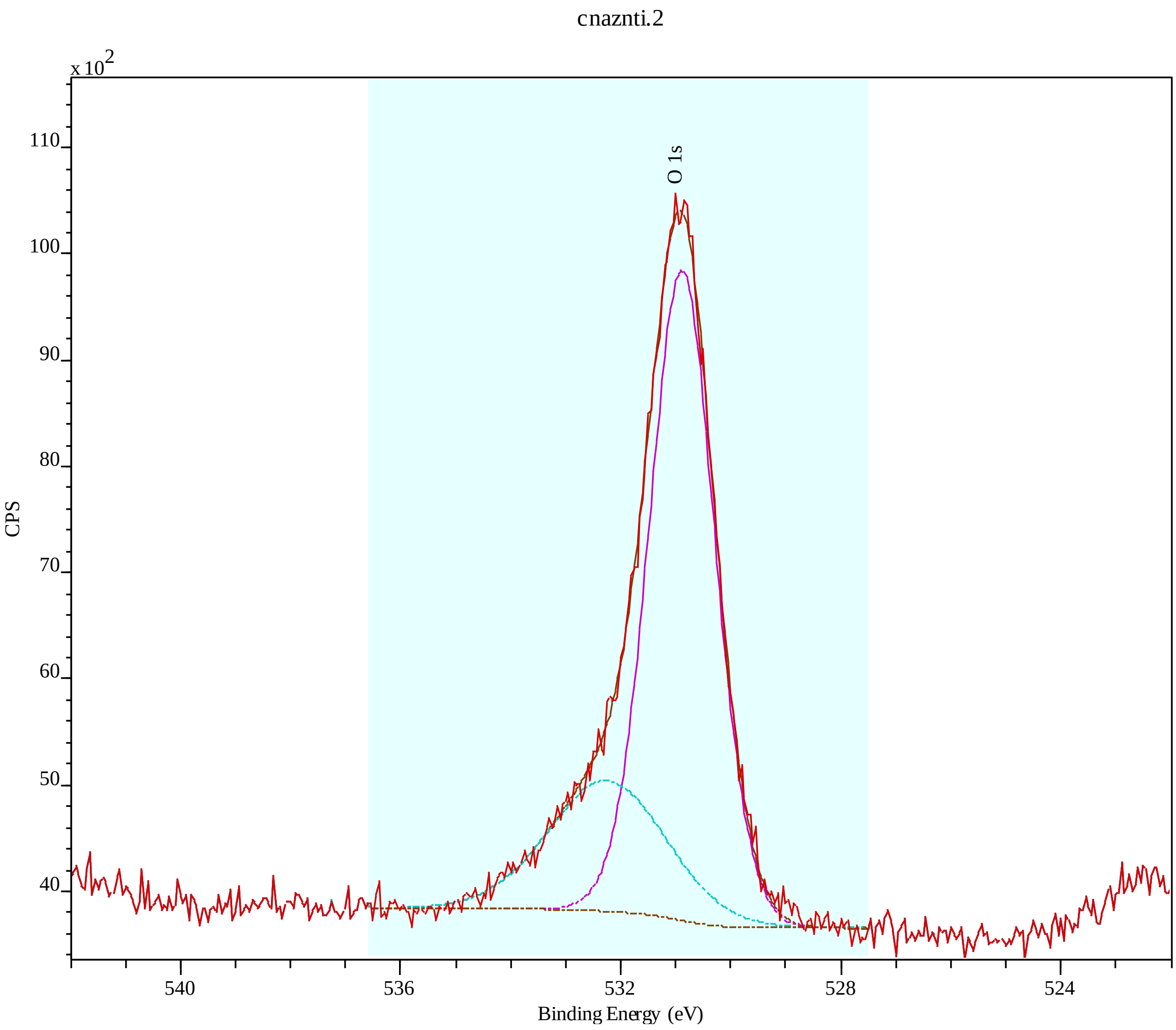

Figure S25. Deconvolution of the O1s peak for CNT-ZnO-TiO_2

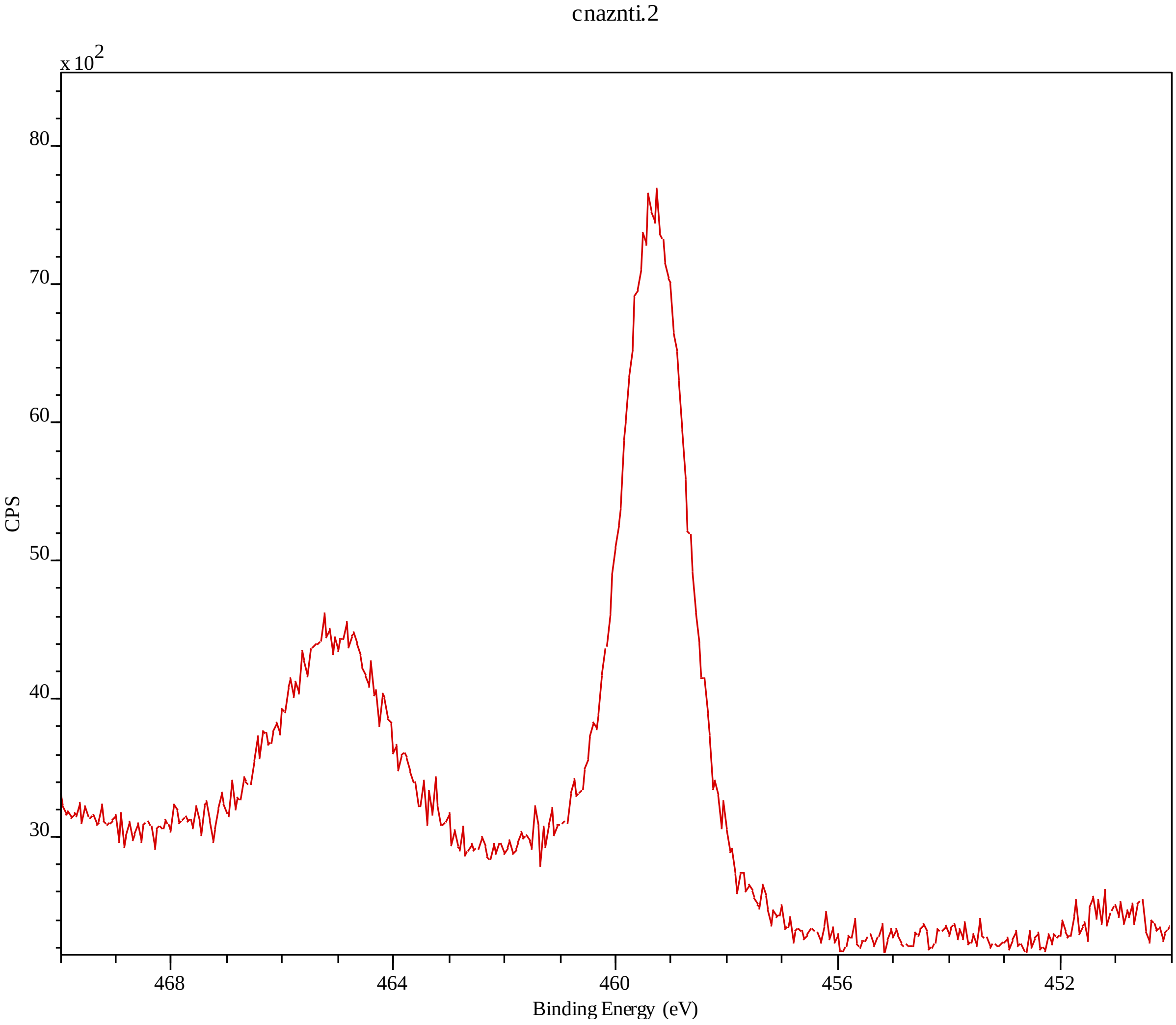

Figure S26. Ti2p peak for CNT-ZnO-TiO_2

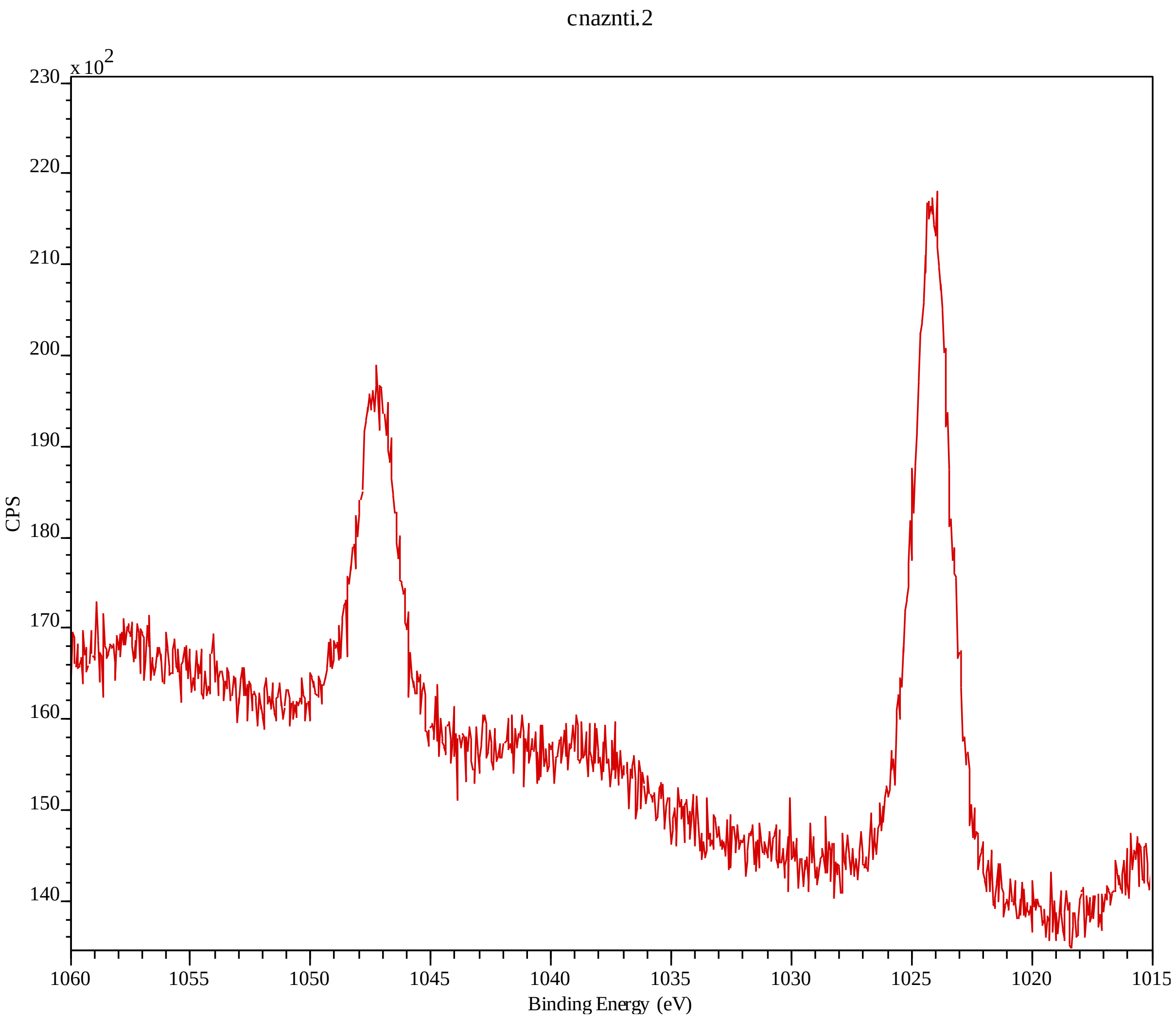

Figure S27. Zn2p peak for CNT-ZnO-TiO_2

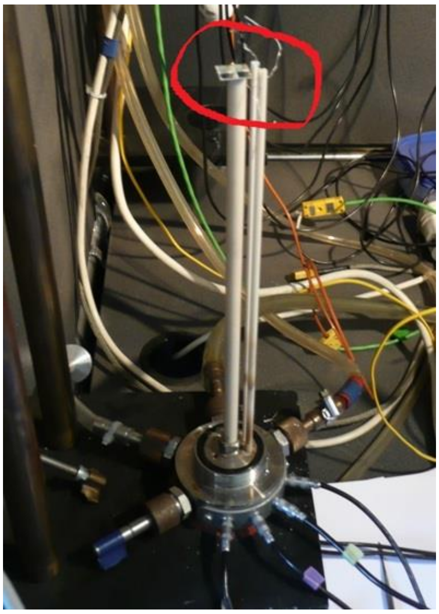

Figure S28. Gas sensor chips on top of ceramic tube, wired in circuit

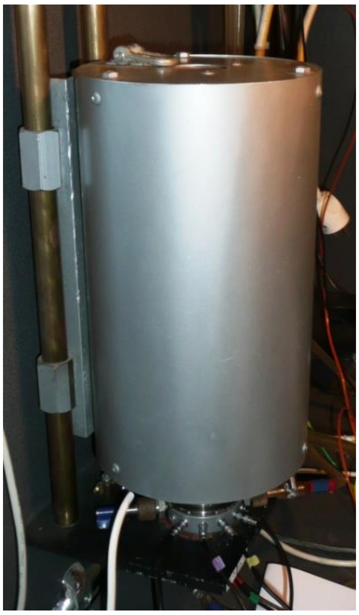

Figure S29. The on-line, temperature and atmosphere controlled furnace mounted on the gas sensor chips
